# Immunomediator expression in human periodontal ligament MSCs varies depending on surface CD146 expression

**DOI:** 10.1038/s41598-026-38627-z

**Published:** 2026-02-22

**Authors:** Christian Behm, Oliwia Miłek, Katharina Schwarz, Alexander Kovar, Oleh Andrukhov

**Affiliations:** Competence Center for Periodontal Research, University Clinic of Dentistry, Medical University of Vienna, Sensengasse 2A, 1090 Vienna, Austria

**Keywords:** Mesenchymal stromal cells, Periodontal ligament, Immunomodulation, CD146 antigen, Immunomediators, Mesenchymal stem cells, Stem-cell research, Periodontitis

## Abstract

Isolating mesenchymal stromal cell (MSC) subpopulations with improved quality based on specific surface markers, such as CD146, is one approach to enhance their therapeutic potential. However, there is limited information on the cytokine-boosted immunomodulatory mechanisms between CD146-expressing and non-expressing MSCs across various inflammatory conditions. This study seeks to address this gap. MSCs from the human periodontal ligament (hPDL-MSCs) were treated with interleukin-(IL)-1β, interferon-(IFN)-γ, or tumor necrosis factor-(TNF)-α, investigating the expression of IDO-1, PD-L1, PTGS-2, and TSG-6 between CD146^+^ and CD146^−^ hPDL-MSCs. Additionally, the expression of the immunomediators was compared between CD146-depleted and -enriched populations, generated by magnetic-bead-sorting. CD146^+^ hPDL-MSCs exhibited a significantly higher proportion of IDO-1^+^ cells, enhanced expression levels in the presence of IL-1β or TNF-α, and higher IL-1β, IFN-γ, or TNF-α-induced PD-L1 protein expression. Conversely, the proportion of TSG-6^+^ hPDL-MSCs was significantly lower in CD146^+^ cells under basal conditions. In CD146-enriched hPDL-MSCs, the immunomediator expression was minimally elevated, with significant differences under specific conditions: a higher PD-L1 protein expression under basal conditions and in the presence of IFN-γ or TNF-α, as well as higher or lower PGE_2_ levels with TNF-α or IL-1β, respectively. These results suggest that the immunomediator expression in hPDL-MSCs alters with different CD146 surface expression. CD146^+^ hPDL-MSCs do not always show higher levels of immunomediator expression. Further research is necessary to isolate hPDL-MSC subpopulations with optimal potential for therapeutic applications.

**Supplementary Information:**

The online version contains supplementary material available at 10.1038/s41598-026-38627-z.

## Introduction

Mesenchymal stromal cells (MSCs) reside in various tissues throughout the human body^1^. First identified in the bone marrow^2^, they have also been isolated from multiple tissues within the oral cavity, including the gingiva^3^, the dental pulp^4^, and the periodontal ligament (PDL)^5^. According to the International Society for Cell and Gene Therapy (ISCT), MSCs express various surface markers (e.g. CD29, CD90, CD73, and CD105) and lack the expression of hematopoietic ones (e.g. CD14, CD19, CD34, CD45, and HLA-DR)^6,7^. Under homeostatic conditions, human periodontal ligament-derived mesenchymal stromal cells (hPDL-MSCs) exist in a dormant, undifferentiated state within the perivascular area of the PDL tissue^8,9^. When tissue damage or inflammation occurs, hPDL-MSCs become activated. They are involved in PDL tissue regeneration and restoring and maintaining homeostatic conditions, mainly by influencing the local inflammatory processes and immune responses^10,11^.

Like MSCs from other tissues, hPDL-MSCs regulate the activity of immune cells of the innate and adaptive immune system^12^. This is accomplished via their primarily immunosuppressively acting, immunomodulatory mechanisms, which include the secretion of paracrine factors and direct interactions between cells. The paracrine mechanisms involve enzymes, including indoleamine-2,3-dioxygenase-1 (IDO-1), prostaglandin G/H synthase 2 (PTGS-2), which produces the soluble immunomediator prostaglandin E_2_ (PGE_2_) and tumor necrosis factor-stimulated gene-6 protein (TSG-6). The direct cell-to-cell contact mechanisms are executed by membrane-bound proteins, including the programmed cell death 1 ligand 1 (PD-L1), encoded by the *CD274* gene^1,12^. The production of these immunomediators and, therefore, the immunomodulatory activities of hPDL-MSCs are highly triggered during inflammation by cytokines, including interleukin-(IL)-1β, interferon-(IFN)-γ, and tumor necrosis factor-(TNF)-α^13,14^. Interestingly, different inflammatory environments promote qualitatively and quantitatively different immunomodulatory properties of MSCs^14^.

The transplantation of ex vivo propagated MSCs isolated from various dental and other tissues is a highly investigated approach for oral and extra-oral tissue regeneration and the treatment of various inflammatory disorders^15,16^. The cytokine-boosted immunosuppressive activities of transplanted MSCs may accelerate the transition from the inflammatory to the proliferative phase during wound healing and, therefore, primarily contribute to their therapeutic effects in vivo^17^. Hence, one approach to increase their therapeutic potential involves the pre-treatment of MSCs with pro-inflammatory cytokines ex vivo^15,18^. Nevertheless, the results of clinical trials are disillusioned and do not contribute to the promising results of basic research studies, which restricts the clinical use of transplanted MSCs. The MSCs’ heterogeneity on the tissue and donor levels and differences in their culture conditions and handling procedures are mainly responsible for the inconsistent outcomes of clinical trials^15,19,20^. Moreover, high variability on the single-cell level with differences within and amongst MSC subpopulations contributes to this inconsistency^1,21–23^. Minimizing this variability is crucial to increasing the reliability of MSCs as a therapeutic tool.

One approach to reduce the MSCs’ heterogeneity is to isolate subpopulations with higher qualities, depending on their surface markers, like CD146/MCAM (melanoma cell adhesion molecule)^24^. In contrast to other MSC markers, like CD29, CD73, CD90, and CD105, the CD146 expression is not consistently expressed within MSC populations^6,7,24–26^. Previous studies reported that CD146-expressing MSCs from various tissues^24,27–32^, including the PDL^33–35^, display a higher differentiation, proliferation, and colony-forming potential. Additionally, a few studies mainly demonstrated stronger immunosuppressive activities of CD146-expressing MSCs from the bone marrow^24^, and umbilical cord^30,36^, against T lymphocytes. Only one study used MSCs from the oral cavity exhibiting the stronger potential of CD146-expressing cells to induce T lymphocyte apoptosis^28^. To our knowledge, no study exists comparing the immunomodulatory abilities of CD146-expressing and non-expressing MSCs isolated from the PDL. Moreover, none of the previous studies investigated the differences in immunomodulatory mechanisms between CD146-expressing and non-expressing MSCs in different inflammatory environments. Hence, getting information on the differences in the immunomodulatory mechanisms between CD146-expressing and non-expressing hPDL-MSCs is essential.

Therefore, this in vitro study aimed to compare the cytokine-boosted immunomodulatory mechanisms between CD146-expressing and non-expressing hPDL-MSCs by investigating the expression/production of IDO-1, *CD274*/PD-L1, PTGS-2/PGE_2_ and *TNFAIP6*/TSG-6. To achieve this aim two approaches were used. In the first approach (see Supplementary Fig. S1a), the cytokine-induced immunomediator production was compared between CD146^−^ and CD146^+^ hPDL-MSCs within heterogenous populations by flow cytometry and appropriate gating. For the second approach (see Supplementary Fig. S1b), magnetic bead sorting was used to generate CD146-depleted and -enriched hPDL-MSC populations, which were cultured separately for cytokine treatment and analysis.

## Results

### Immunomediator expression in CD146^−^ vs. CD146^+^ cells within a hPDL-MSC population under different inflammatory conditions

#### Depending on the cytokine type, IDO-1 protein expression differs between CD146^−^ and CD146^+^ hPDL-MSCs

**Fig. 1:**
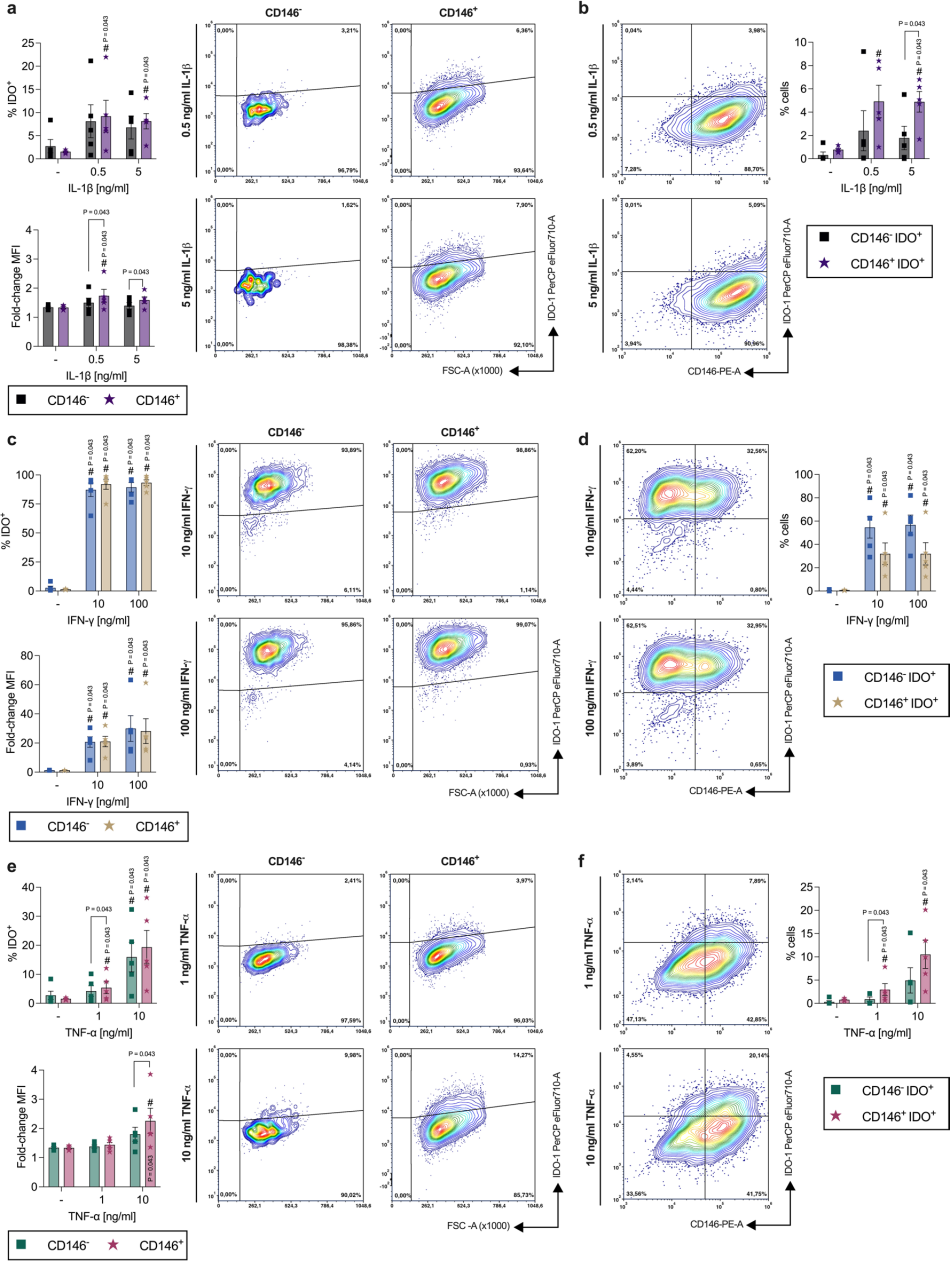
IDO-1 protein expression compared between CD146^−^ and CD146^+^ cells within a heterogenous hPDL-MSC population. After 48 h of incubation in the presence of IL-1β (**a,b**), IFN-γ (**c,d**), or TNF-α (**e,f**), IDO-1/CD146 immunostaining followed by flow cytometry analysis was conducted to compare the % of IDO-1^+^ hPDL-MSCs and the MFI between CD146^−^ and CD146^+^ hPDL-MSCs. The MFI values were normalized to the CD146 single-labeled controls (fold-change MFI = 1) (**a**,** c**,**e**). Additionally, the % of CD146^−^ IDO-1^+α^ and CD146^+^ IDO-1^+^ hPDL-MSCs were determined within the whole hPDL-MSC population (**b**,**d**,**f**). The data were obtained from five independent experiments using hPDL-MSCs from five different donors. They are shown as mean ± standard error of the mean (S.E.M.) with individual data points and as representative dot plots. Statistical significances were obtained by the Wilcoxon Test for pairwise comparison. # p-value < 0.05 is significantly higher compared to the appropriate unstimulated hPDL-MSCs. P-values specified above the connection line describes significantly differences between groups as indicated.

IL-1β (Fig. 1a) enhanced the percentage of IDO-1^+^ cells and the corresponding mean fluorescence intensity (MFI) within CD146^−^ and CD146^+^ hPDL-MSCs, showing a significant increase within the CD146^+^ population. Compared to CD146^−^ cells, CD146^+^ cells displayed a higher percentage of IDO-1^+^ cells (0.5 ng/ml: 1.13-fold, n.s.; 5 ng/ml: 1.20-fold, n.s.). The corresponding IDO-1 MFI were significantly higher in CD146^+^ hPDL-MSCs (0.5 ng/ml: 1.15-fold, *p* = 0.043; 5 ng/ml: 1.14-fold, *p* = 0.043). Within the whole cell population, the rate of CD146^−^ IDO-1^+^ and CD146^+^ IDO-1^+^ hPDL-MSCs was overall lower (Fig. 1b) compared to IDO-1^+^ cells within CD146^−^ and CD146^+^ hPDL-MSCs (Fig. 1a) but was enhanced with both IL-1β concentrations (Fig. 1b). A significant increase was observed for the CD146^+^ IDO-1^+^ populations, additionally showing a higher percentage (0.5 ng/ml: 2.05-fold, n.s.; 5 ng/ml: 2.75-fold, *p* = 0.043) compared to CD146^−^ IDO-1^+^ hPDL-MSCs. Both IFN-γ concentrations (Fig. 1c) significantly increased the percentage of IDO-1^+^ hPDL-MSCs and the MFI within the CD146^−^ and CD146^+^ populations. The percentage of IDO-1^+^ hPDL-MSCs (10 ng/ml: 1.05-fold, n.s.; 100 ng/ml: 1.04-fold, n.s.) within CD146^+^ and CD146^−^ hPDL-MSCs and their corresponding IDO-1 MFI (10 ng/ml: 1.01-fold, n.s.; 100 ng/ml: 0.94-fold, n.s.) showed no differences. The percentages of CD146^−^ IDO-1^+^ and CD146^+^ IDO-1^+^ cells were overall lower within the whole population (Fig. 1d) compared to IDO-1^+^ cells within the CD146^−^ and CD146^+^ populations (Fig. 1c) but were significantly increased with both IFN-γ concentrations (Fig. 1d). A lower percentage of CD146^+^ IDO-1^+^ hPDL-MSCs (10 ng/ml: 0.59-fold, n.s.; 100 ng/ml: 0.56-fold, n.s.) were detected with both IFN-γ concentration.

TNF-α (Fig. 1e) increased the percentage of IDO-1^+^ hPDL-MSCs within CD146^−^ and CD146^+^ hPDL-MSCs in a concentration-dependent manner. A significant increase was detected within the CD146^+^ population with 1 ng/ml TNF-α and in both CD146 populations with 10 ng/ml. The corresponding IDO-1 MFI was enhanced with 10 ng/ml TNF-α, showing a significant increase for the CD146^+^ population. The rate of IDO-1^+^ cells was more elevated within the CD146^+^ population (1 ng/ml: 1.27-fold, *p* = 0.043; 10 ng/ml: 1.21-fold, n.s.), having a significantly higher percentage of IDO-1 cells with 1 ng/ml TNF-α and a significantly higher MFI with 10 ng/ml TNF-α (1.25-fold, *p* = 0.043). The percentages of CD146^−^ IDO-1^+^ and CD146^+^ IDO-1^+^ were overall lower within the whole population (Fig. 1f) compared to IDO-1^+^ cells within CD146^−^ and CD146^+^ hPDL-MSCs (Fig. 1e) but were increased in a concentration-dependent manner. A significant increase was observed for the CD146^+^ IDO-1^+^ hPDL-MSCs. Comparing CD146^−^ IDO-1^+^ with CD146^+^ IDO-1^+^ populations showed a higher percentage of CD146^+^ IDO-1^+^ hPDL-MSCs (1 ng/ml: 3.36-fold, *p* = 0.043; 10 ng/ml: 2.13-fold, n.s.) with a significance detected in the presence of 1 ng/ml TNF-α. These data suggest a different IDO-1 protein expression between CD146^−^ and CD146^+^ populations depending on the cytokine microenvironment.

#### PD-L1 protein expression, but not the rate of PD-L1^+^ hPDL-MSCs, varies between CD146^−^ and CD146^+^ populations

**Fig. 2:**
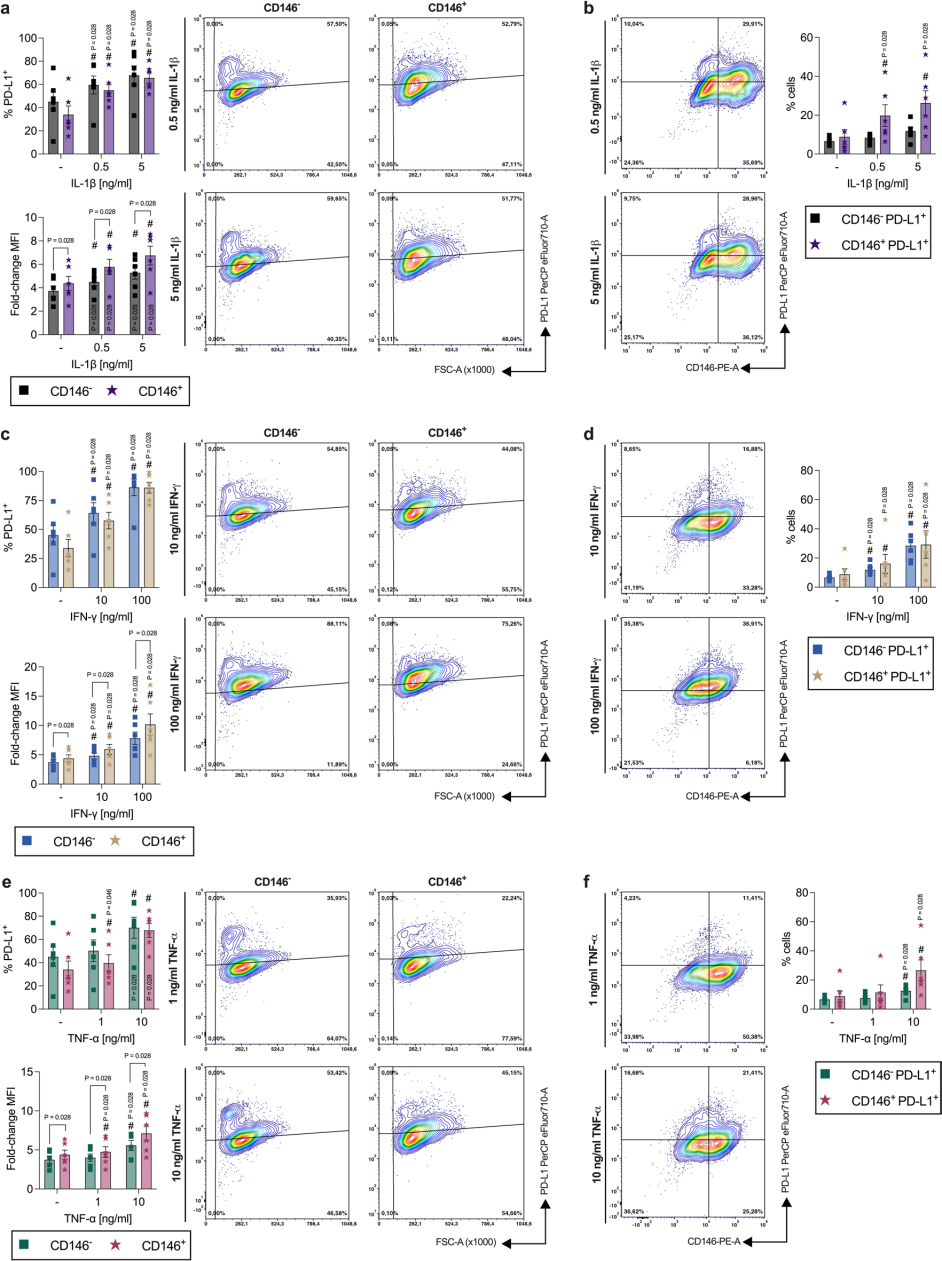
Comparison of PD-L1 protein expression between CD146^−^ and CD146^+^ cells within a heterogenous hPDL-MSC population. After 48 h of incubation with IL-1β (**a,b**), IFN-γ (**c,d**), or TNF-α (**e,f**), PD-L1/CD146 immunostaining and flow cytometry analysis were performed to determine the % of PD-L1^+^ hPDL-MSCs and the MFI within CD146^−^ and CD146^+^ hPDL-MSCs. The MFI values were normalized to the CD146 single-labeled controls (fold-change MFI = 1) (**a**,**c**,**e**). Additionally, the % of CD146^−^ IDO-1^+^ and CD146^+^ IDO-1^+^ hPDL-MSCs were identified within the whole hPDL-MSC population (**b**,**d**,**f**). The data were derived from six independent experiments using hPDL-MSCs from six different patients. They are shown as mean ± S.E.M. with individual data points and as representative dot plots. Statistically significant differences were verified by the Wilcoxon Test for pairwise comparison. ^#^p-value < 0.05 is significantly higher compared to the appropriate unstimulated hPDL-MSCs. P-values specified above the connection line describes significantly differences between groups as indicated.

IL-1β (Fig. 2a) significantly enhanced the percentage of PD-L1^+^ hPDL-MSCs and the corresponding MFIs within the CD146^−^ and CD146^+^ in a concentration-dependent manner. The IL-1β-treated CD146^+^ populations displayed a lower rate of PD-L1^+^ cells with 0.5 ng/ml (0.5 ng/ml: 0.92-fold, n.s.; 5 ng/ml: 0.97-fold, n.s.). In contrast, the PD-L1 MFI were significantly higher within the IL-1β-treated CD146^+^ cells compared to the CD146^−^ populations (0.5 ng/ml: 1.29-fold, *p* = 0.028; 5 ng/ml: 1.28-fold *p* = 0.028). This increase was also observed between CD146^−^ PD-L1^+^ and CD146^+^ PD-L1^+^ hPDL-MSCs within the whole cell population (0.5 ng/ml: 2.37-fold, n.s.; 5 ng/ml: 2.23-fold, n.s.) (Fig. 2b). The percentage of CD146^+^ PD-L1^+^ hPDL-MSCs significantly increased in the presence of IL-1β in a concentration-dependent manner, however, showing an overall lower rate compared to PD-L1^+^ hPDL-MSCs within the CD146^+^ population (Fig. 2a).

In the presence of IFN-γ (Fig. 2c), the rate of PD-L1^+^ hPDL-MSCs and the corresponding MFIs were significantly increased within the CD146^−^ and CD146 ^+^ populations. These rises were concentration-dependent. Within the CD146^+^ populations, a lower percentage of PD-L1^+^ hPDL-MSCs (10 ng/ml: 0.89-fold, n.s.) was observed in the presence of 10 ng/ml IFN-γ. In contrast, the corresponding MFIs were significantly increased within the IFN-γ-treated CD146^+^ populations compared to CD146^−^ cells (10 ng/ml: 1.24-fold, *p* = 0.028; 100 ng/ml: 1.30-fold, *p* = 0.028). The same tendency was detected between CD146^−^ PD-L1^+^ and CD146^+^ PD-L1^+^ hPDL-MSCs within the whole population in the presence of 10 ng/ml IFN-γ (1.35-fold, n.s.) (Fig. 2d). The percentage of both populations significantly increased in the presence of IFN-γ in a concentration-dependent manner, however, showing an overall lower percentage compared to PD-L1^+^ cells within the CD146^−^ and CD146^+^ populations (Fig. 2c).

TNF-α (Fig. 2e) significantly increased the rate of PD-L1^+^ hPDL-MSCs and the corresponding MFI within the CD146^−^ and CD146^+^ populations in a concentration-dependent way. A lower percentage of PD-L1^+^ cells was observed within the CD146^+^ hPDL-MSCs (1 ng/ml: 0.79-fold, n.s.; 10 ng/ml: 0.97-fold, n.s.) in the presence of 1 ng/ml TNF-α. On the contrary, the PD-L1 MFI was significantly elevated within the TNF-α-stimulated CD146^+^ population compared to CD146^−^ cells (1 ng/ml: 1.18-fold, *p* = 0.028; 10 ng/ml: 1.27-fold, *p* = 0.028). The same increase was detected with TNF-α between CD146^−^ PD-L1^+^ and CD146^+^ PD-L1^+^ hPDL-MSCs (1 ng/ml: 1.51-fold, n.s.; 10 ng/ml: 2.12-fold, n.s.) within the whole population (Fig. 2f). The percentage of both populations significantly increased in the presence of 10 ng/ml TNF-α, however, displaying an overall lower rate compared to PD-L1^+^ cells within the CD146^−^ and CD146^+^ populations (Fig. 2e). Together, these data indicate a higher PD-L1 protein expression in CD146^+^ cells compared to CD146^−^ populations, but not a higher rate of PD-L1^+^ hPDL-MSCs within these two populations.

#### CD146^−^ and CD146^+^ hPDL-MSC populations show similar PTGS-2 protein expression

**Fig. 3:**
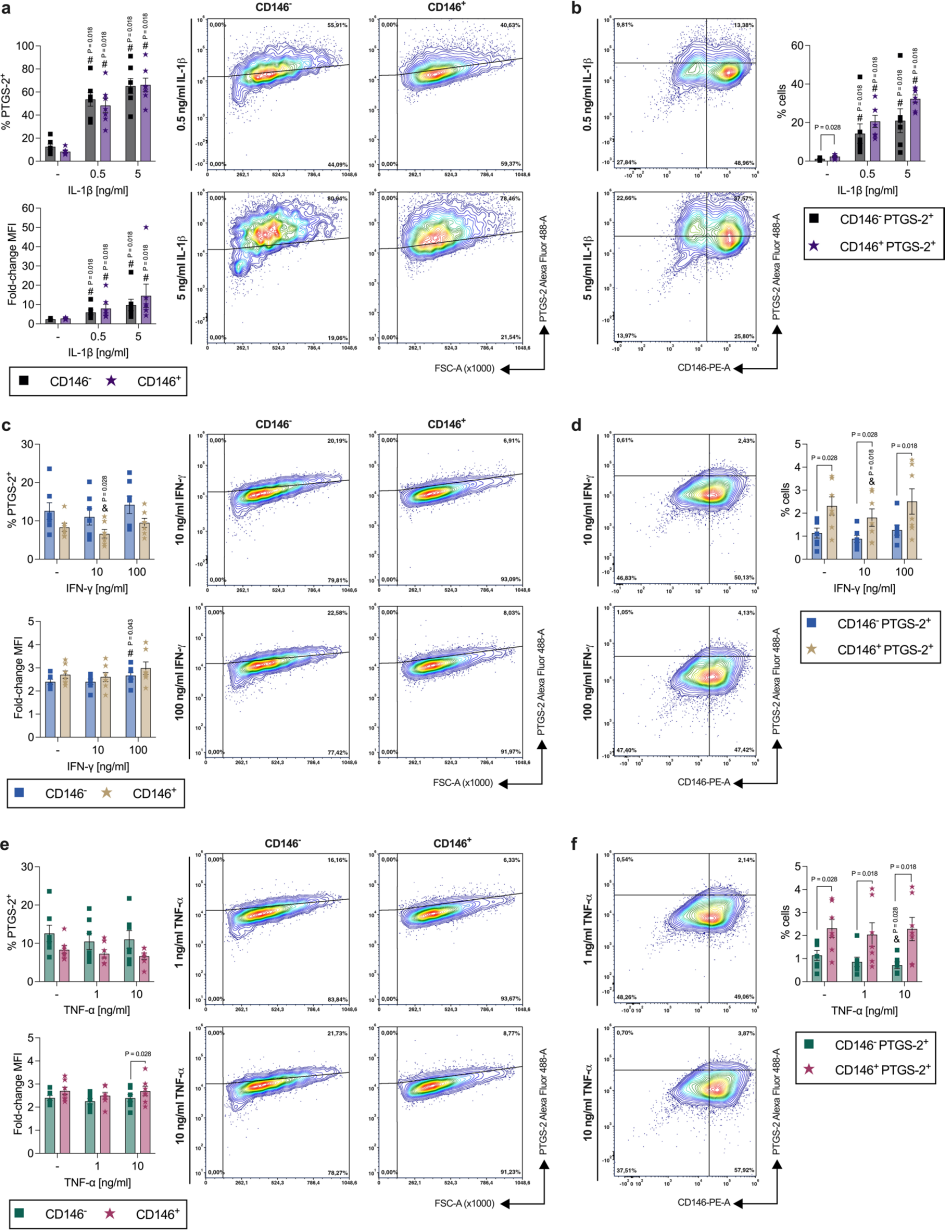
Comparison of PTGS-2 protein expression between CD146^−^ and CD146^+^ cells within a heterogenous hPDL-MSC population. After 48 h of incubation with IL-1β (**a,b**), IFN-γ (**c,d**), or TNF-α (**e,f**), PTGS-2/CD146 immunostaining and flow cytometry analysis were executed to determine the % of PTGS-2^+^ hPDL-MSCs and the MFI within CD146^−^ and CD146^+^ hPDL-MSCs. The MFI values were normalized to the CD146 single-labeled controls (fold-change MFI = 1) (**a**,**c**,**e**). Additionally, the % of CD146^−^ PTGS-2^+^ and CD146^+^ PTGS-2^+^ hPDL-MSCs were identified within the whole hPDL-MSC population (**b**,**d**,**f**). The data were derived from seven individual experimental repetitions using hPDL-MSCs from seven different individuals. The data are shown as mean ± S.E.M. with individual data points and as representative dot plots. Statistically significant differences were verified by the Wilcoxon Test for pairwise comparison. ^#^p-value < 0.05 is significantly higher compared to the appropriate unstimulated hPDL-MSCs. ^&^p-value < 0.05 is significantly lower compared to the appropriate unstimulated hPDL-MSCs. P-values specified above the connection line describes significantly differences between groups as indicated.

IL-1β (Fig. 3a) significantly enhanced the percentage of PTGS-2^+^ hPDL-MSCs and the associated MFI within the CD146^−^ and CD146^+^ populations in a concentration-dependent way. A lesser rate of PTGS-2^+^ hPDL-MSCs was observed within the CD146^+^ population with 0.5 ng/ l IL-1β (0.5 ng/ml: 0.90-fold, n.s.). In contrast, IL-1β-treated CD146^+^ cells displayed higher PTGS-2 MFI compared to CD146^−^ hPDL-MSCs (0.5 ng/ml: 1.34-fold, n.s.; 5 ng/ml: 1.49-fold, n.s.). The same tendency was also detected for the CD146^−^ PTGS-2^+^ and CD146^+^ PTGS-2^+^ populations within the whole cell population in the presence of IL-1β (0.5 ng/ml: 1.45-fold, n.s.; 5 ng/ml: 1.54-fold, n.s.) and with a significantly higher rate on CD146^+^ PTGS-2^+^ hPDL-MSCs (2.04-fold, *p* = 0.028) in the absence of any cytokine (Fig. 3b). Although IL-1β significantly increased the percentage of CD146^−^ PTGS-2^+^ and CD146^+^ PTGS-2^+^ hPDL-MSCs in a concentration-dependent manner, the overall rate was lower compared to the percentage of PTGS-2^+^ cells within the CD146^−^ and CD146^+^ populations (Fig. 3a).

IFN-γ (Fig. 3c,d) and TNF-α (Fig. 3e,f) did not cause any increase in PTGS-2 protein expression at all. The percentage of PTGS-2^+^ cells within the CD146^−^ and CD146^+^ populations, the associated MFI (Fig. 3c,e), as well as the percentage of CD146^−^ PTGS-2^+^ and CD146^+^ PTGS-2^+^ hPDL-MSCs (Fig. 3d,f), were comparable to the appropriate unstimulated hPDL-MSCs. Hence, the biological importance of the significance between the different populations in the presence of IFN-γ (Fig. 3c,d) or TNF-α (Fig. 3e,f) is inconsequential. These data indicate similar IL-1β-induced PTGS-2 expression between CD146^−^ and CD146^+^ hPDL-MSCs.

#### CD146^+^ population reveals a lower percentage of TSG-6^+^ hPDL-MSCs without any exogenous cytokine

**Fig. 4:**
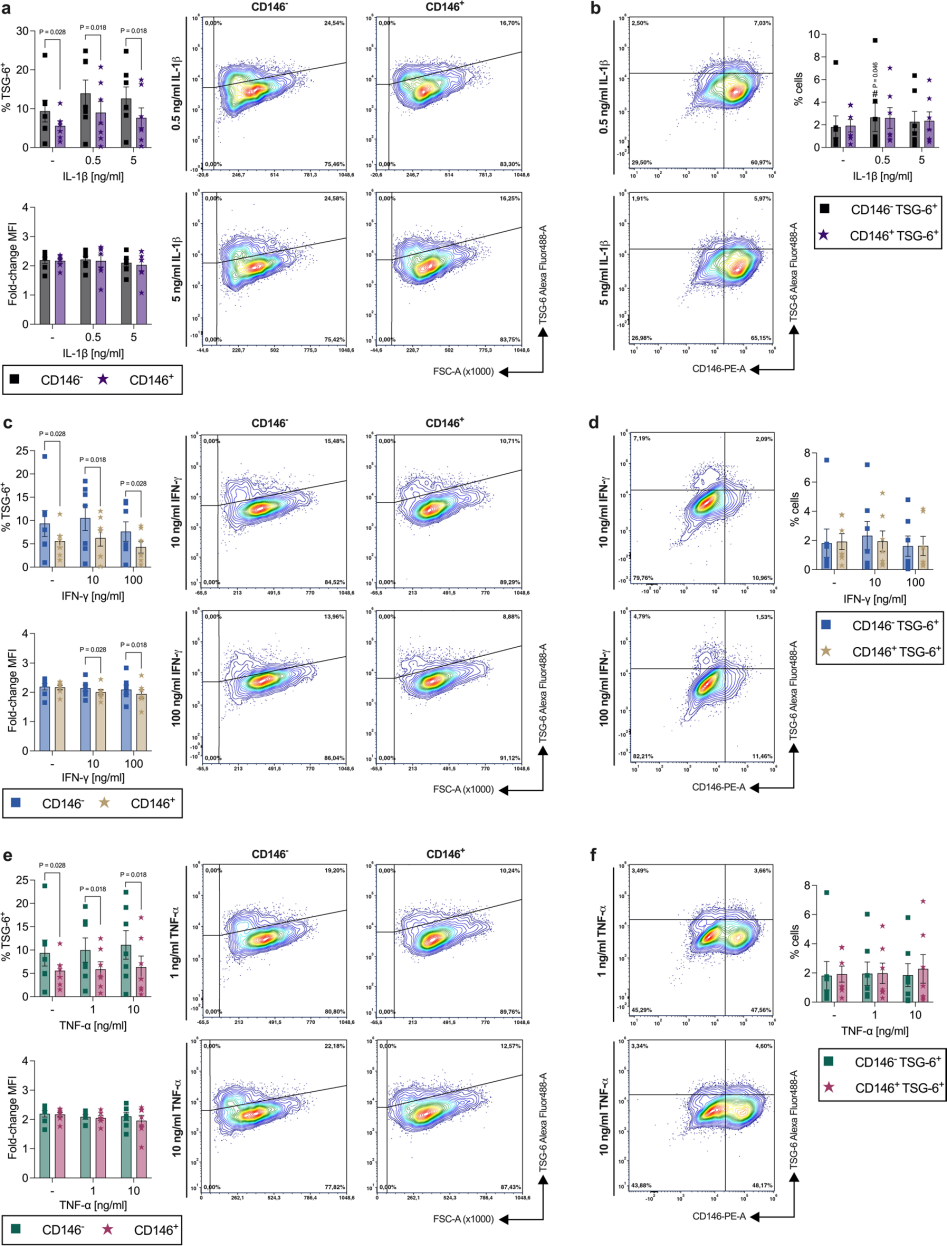
TSG-6 protein expression compared between CD146^−^ and CD146^+^ cells within a heterogenous hPDL-MSC population. After 48 h of incubation with IL-1β (**a,b**), IFN-γ (**c,d**), or TNF-α (**e,f**), TSG-6/CD146 immunostaining and flow cytometry analysis were performed to determine the % of TSG-6^+^ hPDL-MSCs and the MFI within CD146^−^ and CD146^+^ hPDL-MSCs. The MFI values were normalized to the CD146 single-labeled controls (fold-change MFI = 1) (**a**,**c**,**e**). Additionally, the % of CD146^−^ TSG-6^+^ and CD146^+^ TSG-6^+^ hPDL-MSCs were identified within the whole hPDL-MSC population (**b**,**d**,**f**). The data were obtained from seven independent experiments using hPDL-MSC from seven different donors. The data are presented as mean ± S.E.M. with individual data points and as representative dot plots. Statistically significant differences were determined by the Wilcoxon Test for pairwise comparison. ^#^p-value < 0.05 is significantly higher compared to the appropriate unstimulated hPDL-MSCs. P-values specified above the connection line describes significantly differences between groups as indicated.

In the absence of any exogenous cytokines, CD146^+^ populations possessed a significantly lower (0.60-fold, *p* = 0.028) percentage of TSG-6^+^ hPDL-MSCs (Fig. 4a,c,e). The corresponding MFI (0.99-fold, n.s.) and the percentage of CD146^−^ TSG-6^+^ and CD146^+^ TSG-6^+^ populations (1.1-fold, n.s.) displayed no differences (Fig. 4b,d,f). Adding IL-1β (Fig. 4a,b), IFN-γ (Fig. 4c,d), or TNF-α (Fig. 4e,f) did not influence either the percentage of TSG-6^+^ hPDL-MSCs within the CD146^−^ and CD146^+^ populations and the corresponding MFI, or the percentage of CD146^−^ TSG-6^+^ and CD146^+^ TSG-6^+^ populations. Therefore, the biological importance of the detected differences in the rate of TSG-6^+^ hPDL-MSCs between the CD146^−^ and CD146^+^ populations with IL-1β (Fig. 4a), IFN-γ (Fig. 4c), or TNF-α (Fig. 4e) are inconsequential. Together, these data suggest a lower number of hPDL-MSCs within the CD146^+^ populations that express TSG-6 on the protein level, and that these differences between CD146^−^ and CD146^+^ are independent of the present cytokine.

### Immunomediator expression in CD146-depleted vs. CD146-enriched hPDL-MSC populations under different cytokine environments

Isolated hPDL-MSCs displayed a heterogenous CD146 expression, including CD146^−^ and CD146^+^ cells (see Supplementary Fig. S2a online). Magnetic bead sorting resulted in a CD146-depletion/enrichment. The CD146-depleted and -enriched hPDL-MSC populations predominantly consisted of CD146^−^ and CD146^+^ hPDL-MSCs without completely excluding CD146^+^ or CD146^−^ cells, and therefore naming them CD146-depleted or -enriched hPDL-MSC populations, respectively (see Supplementary Fig. S2a). Nevertheless, the percentage of CD146^+^ hPDL-MSCs was significantly higher in the CD146-enriched populations compared to the CD146-depleted cells (see Supplementary Fig. 2b). Together, this indicates a successful enrichment of CD146^+^ hPDL-MSCs.

#### CD146-depleted and -enriched hPDL-MSC populations show similar IDO-1 expression and enzymatic activity

**Fig. 5:**
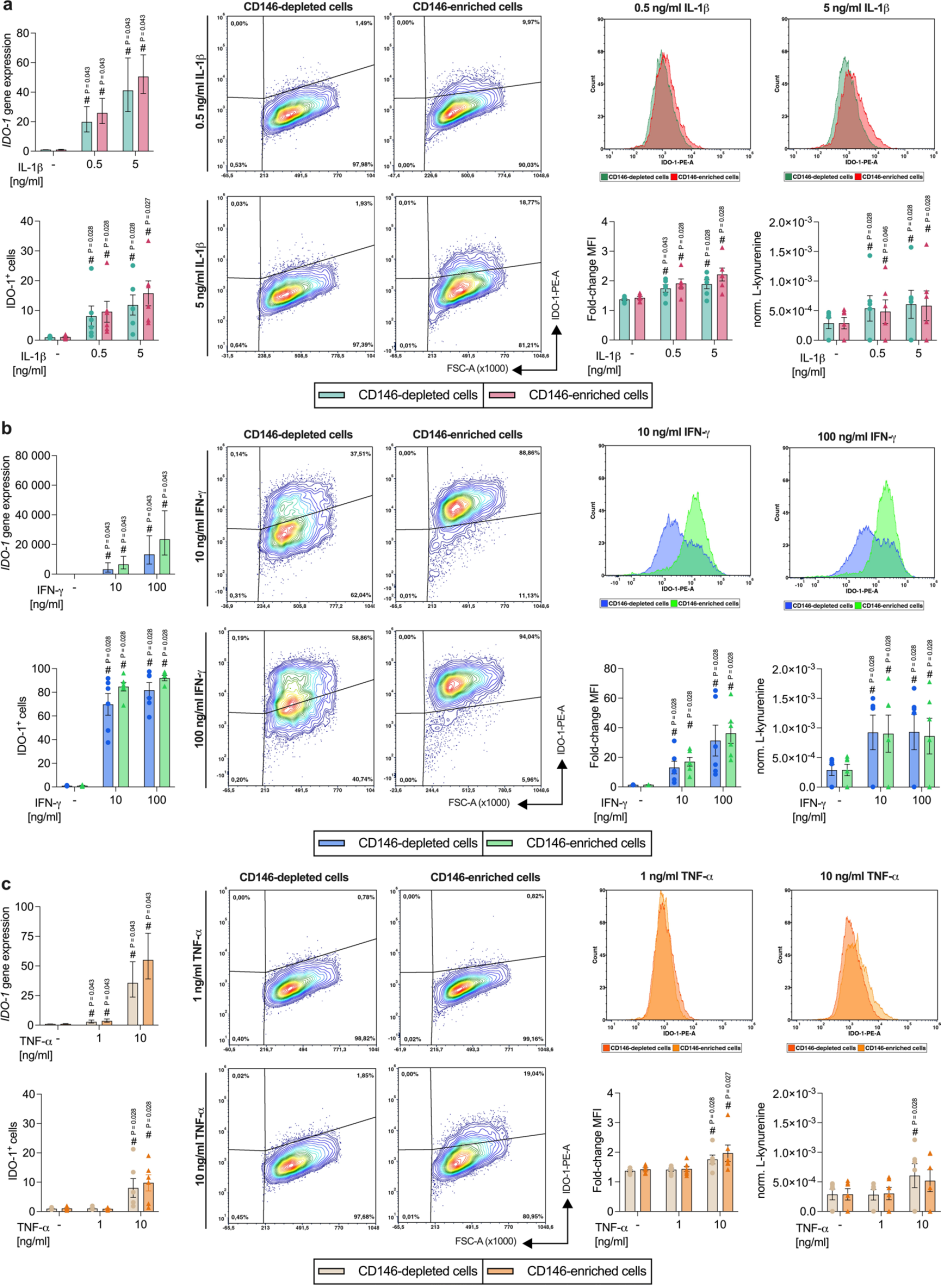
IDO-1 expression compared between CD146-depleted and -enriched hPDL-MSCs. CD146-depleted and -enriched hPDL-MSCs were stimulated with IL-1β (**a**), IFN-γ (**b**), or TNF-α (**c**). After 48 h of incubation, *IDO-1* gene expression was investigated using qPCR, determining the n-fold *IDO-1* expression compared to the appropriate unstimulated controls (n-fold expression = 1). The percentage of IDO-1^+^ hPDL-MSCs and the fold-change in the MFI of the whole cell population compared to the unlabeled CD146-depleted or -enriched hPDL-MSCs was calculated by flow cytometry analysis. Additionally, the L-kynurenine concentrations were measured photometrically and normalized to the appropriate cell number to define the IDO-1 enzymatic activity. The data were obtained from five or six independent repetitions using hPDL-MSCs from five or six different patients for qPCR or protein analysis, respectively. The data are presented as mean ± S.E.M. For flow cytometry analysis and enzymatic activity assay, single data points are additionally presented, and the % of IDO-1^+^ hPDL-MSCs and MFI are shown as representative dot plots and histograms, respectively. Statistical significances were obtained by using the Wilcoxon Test for pairwise comparison. # p-value < 0.05 is significantly higher compared to the unstimulated CD146-depelted or -enriched hPDL-MSC population.

IL-1β (Fig. 5a) significantly increased IDO-1 gene expression, the percentage of IDO-1^+^ hPDL-MSCs, the IDO-1 MFI, and the enzymatic activity. This rise was concentration-dependent and observed in CD146-depleted and -enriched populations. A higher IDO-1 gene (0.5 ng/ml: 1.31-fold n.s.; 5 ng/ml; 1.23-fold, n.s.) and protein (% IDO-1^+^, 0.5 ng/ml: 1.18-fold, n.s., 5 ng/ml: 1.33-fold, n.s.; MFI, 0.5 ng/ml: 1.10-fold, n.s., 5 ng/ml: 1.17-fold, n.s.) expression was observed in the CD146-enriched hPDL-MSCs. In contrast, a lower (0.5 ng/ml: 0.90-fold, n.s.; 5 ng/ml: 0.96-fold, n.s.) enzymatic activity was detected in the CD146-enriched population in the presence of 0.5 ng/ml IL-1β.

IFN-γ (Fig. 5b) significantly enhanced IDO-1 gene expression, the percentage of IDO-1^+^ hPDL-MSCs, the IDO-1 MFI, and the enzymatic activity in a concentration-dependent manner. This increase in IDO-1 expression was witnessed in CD146-depleted and -enriched hPDL-MSCs. CD146-enriched populations displayed higher IDO-1 gene (10 ng/ml: 2.03-fold, n.s.; 100 ng/ml: 1.77-fold, n.s.) and protein (% IDO-1^+^, 10 ng/ml: 1.21-fold, n.s., 100 ng/ml: 1.12-fold, n.s.; MFI, 10 ng/ml: 1.30-fold, n.s., 100 ng/ml: 1.16-fold, n.s.) expression. In contrast, the IDO-1 enzymatic activity was similar (10 ng/ml: 0.98-fold, n.s) or marginally lower (100 ng/ml: 0.93-fold, n.s.) in CD146-enriched hPDL-MSCs.

TNF-α (Fig. 5c) treated CD146-depleted and -enriched hPDL-MSCs showed a significant increase in IDO-1 gene expression in a concentration-dependent manner. The IDO-1 protein levels significantly increased with 10 ng/ml TNF-α in both populations, whereas the enzymatic activity was improved only in CD146-depleted hPDL-MSCs. Differences between CD146-depleted and -enriched hPDL-MSCs were observed with 10 ng/ml TNF-α, showing higher IDO-1 gene (1.54-fold, n.s.) and protein (% IDO-1^+^, 1.22-fold, n.s.; MFI, 1.12-fold, n.s.) expression and a lower (0.86-fold, n.s.) enzymatic activity in CD146-enriched populations. These data point to comparable cytokine-induced IDO-1 expression and enzymatic activities in CD146-depleted and -enriched hPDL-MSCs.

#### Depending on the cytokine type, PD-L1 protein expression differs between CD146-depleted and -enriched hPDL-MSCs

**Fig. 6:**
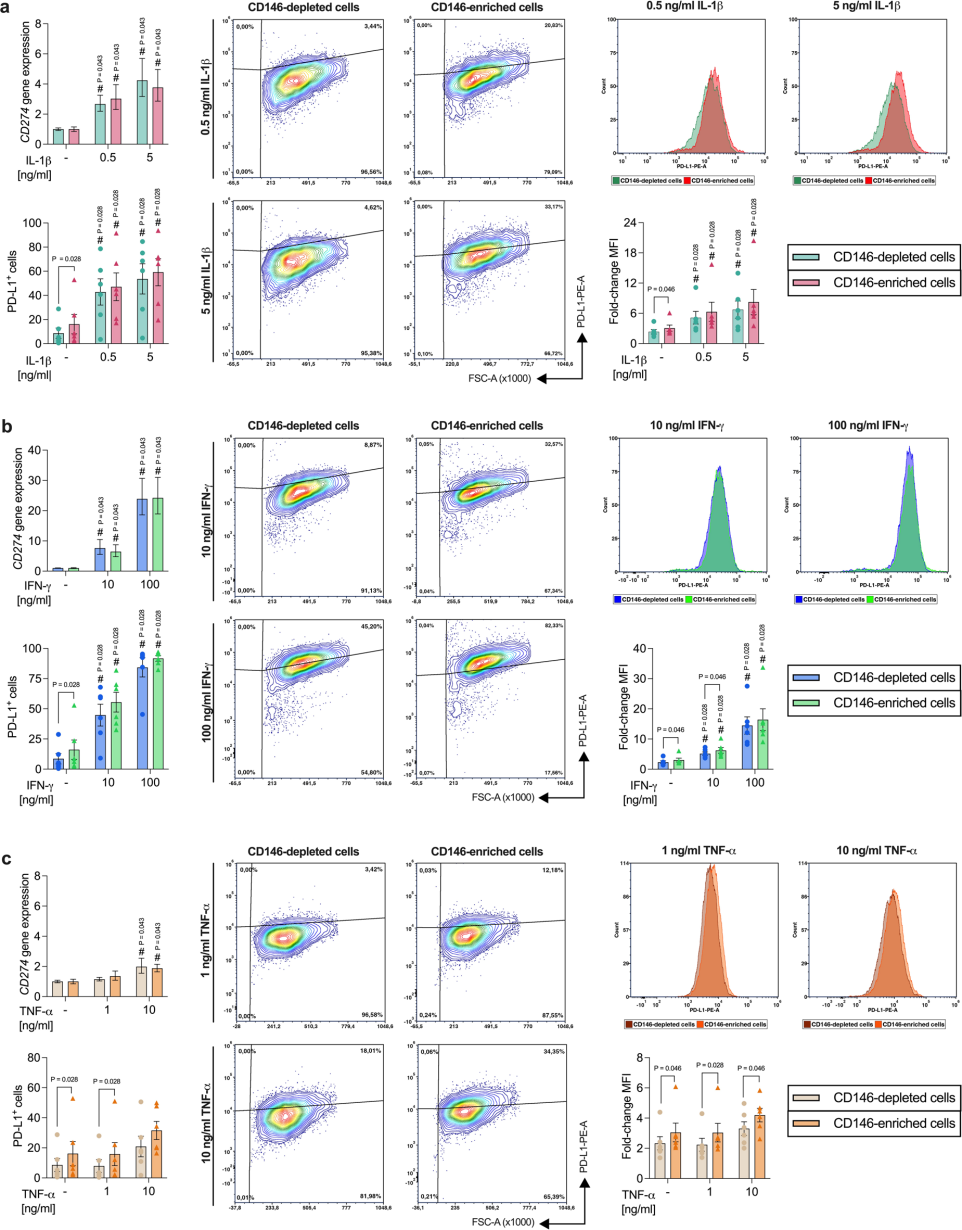
*CD274*/PD-L1 expression compared between CD146-depleted and -enriched hPDL-MSCs. CD146-depleted and -enriched hPDL-MSCs were treated with IL-1β (**a**), IFN-γ (**b**), or TNF-α (**c**). After 48 h of incubation, *CD274* gene expression was determined by qPCR, calculating the n-fold *CD274* expression compared to the appropriate untreated hPDL-MSCs (n-fold expression = 1). The percentage of PD-L1^+^ hPDL-MSCs and the fold-change in the MFI of the whole population compared to the unlabeled CD146-depleted or -enriched hPDL-MSCs was calculated by flow cytometry analysis. The data were obtained from five or six independent repetitions using hPDL-MSCs from five or six different individuals for qPCR or immunostaining, respectively and are shown as mean ± S.E.M. The flow cytometry data are additionally presented as individual data points and representative dot plots and histograms demonstrate the % of PD-L1^+^ hPDL-MSCs and the MFI, respectively. Statistical significances were obtained by the Wilcoxon Test for pairwise comparison. # p-value < 0.05 is significantly higher compared to the unstimulated CD146-depleted or -enriched hPDL-MSC population. *p-value < 0.05 is significantly different between CD146-depleted and -enriched hPDL-MSC population. P-values specified above the connection line describes significantly differences between CD146-depleted and -enriched hPDL-MSCs.

IL-1β (Fig. 6a) significantly enhanced *CD274* gene expression, the percentage of PD-L1^+^ hPDL-MSCs, and the corresponding MFI. This gain was concentration-dependent and detected in CD146-depleted and -enriched hPDL-MSCs. A 1.13-fold (n.s.) higher *CD274* gene expression was detected in the CD146-enriched population with 0.5 ng/ml IL-1β, whereas with 5 ng/ml the *CD274* gene expression was 0.89-fold (n.s.) lower in CD146-enriched hPDL-MSCs. The percentage of PD-L1^+^ hPDL-MSCs (0.5–5 ng/ml: 1.10-fold, n.s.) and its MFI (0.5–5 ng/ml: 1.22-fold, n.s.) were higher in the IL-1β-treated CD146-enriched population.

IFN-γ (Fig. 6b) significantly boosted the *CD274* gene and PD-L1 protein expression. This increase was concentration-dependent and detected in CD146-depleted and -enriched populations. The CD146-enriched populations exhibited a 0.85-fold (n.s.) lower *CD274* gene expression with 10 ng/ml IFN-γ compared to CD146-depleted hPDL-MSCs, whereas no differences were observed with 100 ng/ml IFN-γ. On the protein level, IFN-γ-treated CD146-enriched hPDL-MSCs displayed a higher percentage of PD-L1^+^ hPDL-MSCs (10 ng/ml: 1.24, n.s.; 100 ng/ml: 1.09-fold, n.s.) and MFI (10 ng/ml: 1.21-fold, *p* = 0.046; 100 ng/ml: 1.13-fold, n.s.), showing a significant higher fold-change in the MFI with 10 ng/ml IFN-γ.

With 10 ng/ml TNF-α (Fig. 6c) a significant increase in *CD274* gene expression was observed in both populations. No significant increase in PD-L1 protein expression was detected. The CD146-enriched population featured a 1.18-fold (n.s.) higher but a 0.95-fold (n.s.) lower *CD274* gene expression with 1 ng/ml and 10 ng/ml TNF-α, respectively. The percentage of PD-L1^+^ hPDL-MSCs (1 ng/ml: 2.00-fold, *p* = 0.028, 10 ng/ml: 1.51-fold, n.s.) and its MFI (1 ng/ml: 1.36-fold, *p* = 0.028; 10 ng/ml: 1.27-fold, *p* = 0.046) were higher in the CD146-enriched population compared to the depleted one. A significant increase was observed for the percentage of PD-L1^+^ hPDL-MSCs with 1 ng/ml TNF-α and for the fold-change of the MFI with both TNF-α concentrations. Additionally, in the absence of any cytokine, the basal PD-L1 protein expression was significantly higher (% of PD-L1^+^, 1.87-fold, *p* = 0.028; MFI, 1.31-fold, *p* = 0.046) in the CD146-enriched population compared to CD146-depleted hPDL-MSCs. Together, these data indicate that CD146-enriched hPDL-MSCs possess a higher basal PD-L1 protein expression than CD146-depleted cells and that this difference is influenced by the cytokine microenvironment.

#### Depending on the cytokine environment, PGE_2_ levels vary between CD146-depleted and -enriched hPDL-MSCs

**Fig. 7:**
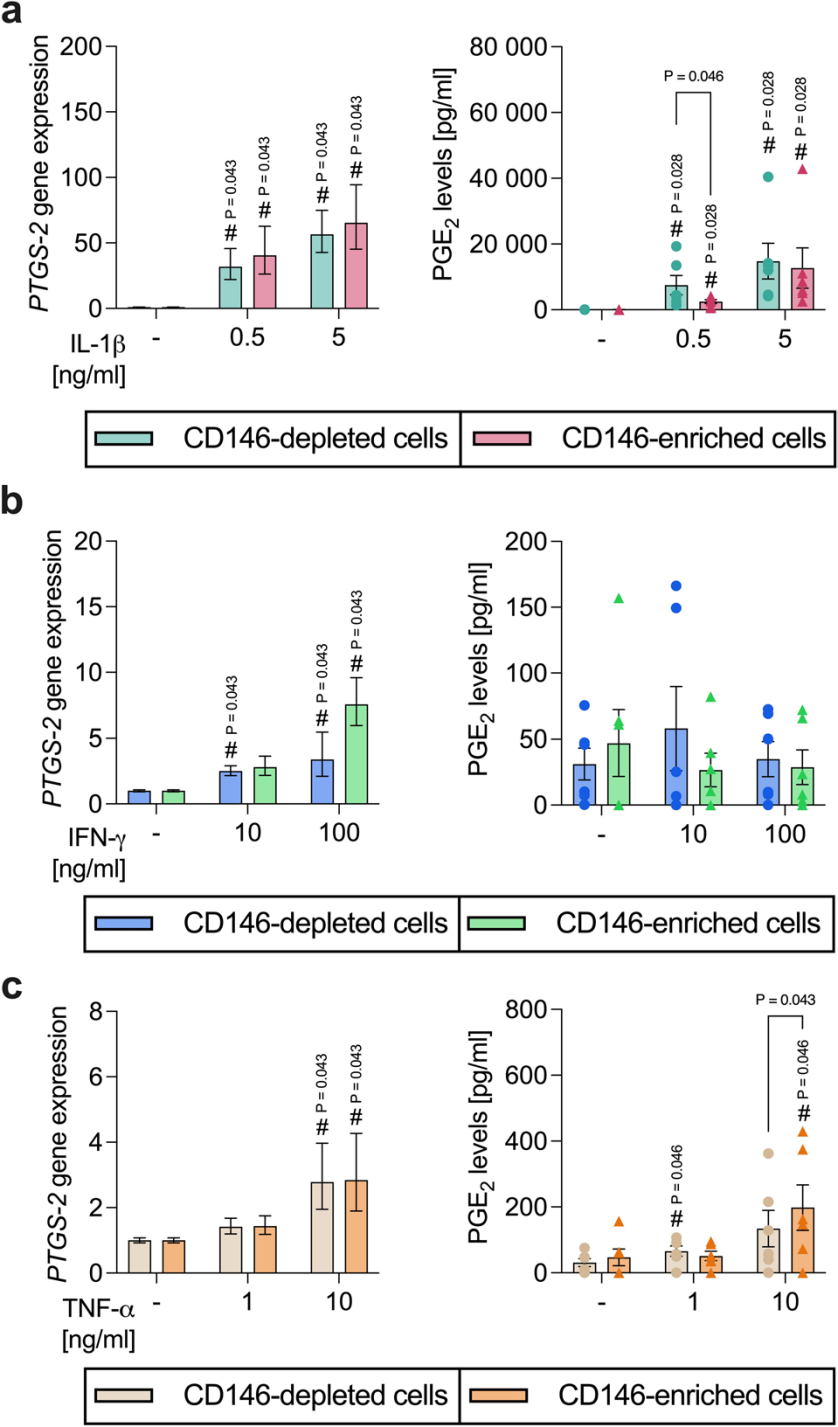
*PTGS-2* gene expression and PGE_2_ secretion compared between CD146-depleted and -enriched hPDL-MSCs. CD146-depleted and -enriched hPDL-MSCs were treated with IL-1β (**a**), IFN-γ (**b**), or TNF-α (**c**). After 48 h of incubation, *PTGS-2* gene expression was identified by qPCR, determining the n-fold *PTGS-2* expression compared to the appropriate untreated hPDL-MSCs (n-fold expression = 1). The level of secreted PGE_2_ [pg/ml] was determined by ELISA in the harvested conditioned media. The data were received from five or six independent repetitions with hPDL-MSCs isolated from five or six different individuals for qPCR or PGE_2_ parameter assay, respectively, and are demonstrated as mean ± S.E.M. Additionally, individual data points are presented for the PGE_2_ secretion levels. The Wilcoxon Test for pairwise comparison was used to determine statistically significant differences. ^#^p-value < 0.05 is significantly higher compared to the unstimulated CD146-depelted or -enriched hPDL-MSC population. P-values specified above the connection line describe significant differences between CD146-depleted and -enriched hPDL-MSCs.

 IL-1β (Fig. 7a) caused a significant increase in *PTGS-2* and PGE_2_ levels in a concentration-dependent manner. This increase was observed in CD146-depleted and -enriched hPDL-MSCs. A higher (0.5 ng/ml: 1.27-fold, n.s.; 5 ng/ml: 1.15-fold, n.s.) *PTGS-2* gene expression was observed in CD146-enriched hPDL-MSCs. In contrast, the PGE_2_ level was lower (0.5 ng/ml: 0.34-fold, *p* = 0.046; 5 ng/ml: 0.86-fold, n.s.) in the CD146-enriched populations compared to the CD146-depleted hPDL-MSCs, showing a significant difference with 0.5 ng/ml IL-1β.

IFN-γ (Fig. 7b) significantly triggered the *PTGS-2* gene expression concentration-dependent. A significant increase in *PTGS-2* gene expression was observed for CD146-depleted and -enriched hPDL-MSCs, except for CD146-enriched hPDL-MSCs with 10 ng/ml IFN-γ. In contrast, IFN-γ had no impact on the PGE_2_ levels. The IFN-γ-triggered *PTGS-2* gene expression was higher (10 ng/ml: 1.12-fold, n.s.; 100 ng/ml: 2.23-fold, n.s.) in CD146-enriched populations.

The *PTGS-2* gene expression and PGE_2_ levels were also boosted by TNF-α (Fig. 7c) in a concentration-dependent manner. A significant increase in *PTGS-2* gene expression was detected with 10 ng/ml TNF-α for CD146-depleted and -enriched hPDL-MSCs, whereas the PGE_2_ level was significantly enhanced in the CD146-depleted and -enriched populations with 1 ng/ml and 10 ng/ml TNF-α, respectively. On the gene level, no differences between CD146-depleted and -enriched hPDL-MSCs were observed. In CD146-enriched hPDL-MSCs, the PGE_2_ level was significantly higher (1.47-fold, *p* = 0.043) and 0.78-fold (n.s.) lower with 10 ng/ml and 1 ng/ml TNF-α, respectively. These data indicate differences in the PGE_2_ levels between CD146-depleted and -enriched hPDL-MSCs depending on the cytokine environment.

#### CD146-depleted and -enriched hPDL-MSC populations display comparable TNFAIP6 gene and TSG-6 protein expression

**Fig. 8:**
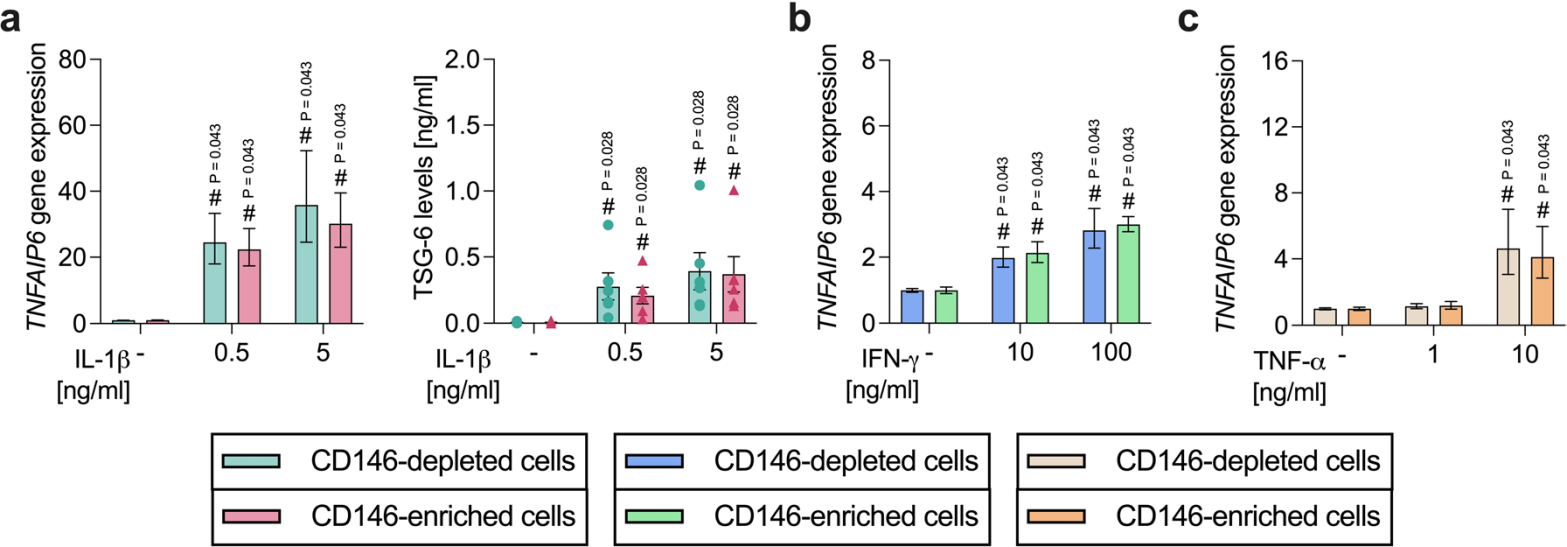
*TNFAIP*/TSG-6 expression compared between CD146-depleted and -enriched hPDL-MSCs. CD146-depleted and -enriched hPDL-MSCs were treated with IL-1β (**a**), IFN-γ (**b**), or TNF-α (**c**). After 48 h of incubation, *TNFAIP6* gene expression was determined by qPCR, calculating the n-fold *TNFAIP6* expression compared to the appropriate untreated hPDL-MSCs (n-fold expression = 1). The TSG-6 levels [ng/ml] were determined in the harvested conditioned media from the untreated and IL-1β stimulated samples (**a**) using ELISA. The data were received from five or six independent experiments using hPDL-MSCs from five or six patients for qPCR or TSG-6 ELISA, respectively, and are presented as mean ± S.E.M. The TSG-6 concentration levels are additionally shown as individual data points. The Wilcoxon Test for pairwise comparison was used to determine statistically significant differences. ^#^p-value < 0.05 is significantly higher compared to the unstimulated CD146-depelted or -enriched hPDL-MSC population.

IL-1β (Fig. 8a) caused a significantly higher *TNFAIP6* gene expression compared to the unstimulated hPDL-MSCs. This increase demonstrated a concentration dependency and was revealed in CD146-depleted and -enriched hPDL-MSCs. The same significant increase was also observed for the TSG-6 protein levels. *TNFAIP6* gene expression (0.5 ng/ml: 0.91-fold, n.s.; 5 ng/ml: 0.84-fold, n.s.) and the TSG-6 levels (0.5 ng/ml: 0.76-fold, n.s.; 5 ng/ml: 0.94-fold, n.s.) were reduced in CD146-enriched populations compared to CD146-depleted hPDL-MSCs.

The *TNFAIP6* gene expression was significantly enhanced with IFN-γ (Fig. 8b) in a concentration-dependent manner. This enhancement was detected in CD146-depleted and -enriched hPDL-MSCs with comparable TNFAIP6 gene expression levels in the two different populations (10 ng/ml: 1.08-fold, n.s.; 100 ng/ml: 1.07, n.s.). IFN-γ-induced TSG-6 protein levels were not detectable.

TNF-α (Fig. 8c) significantly triggered the *TNFAIP6* gene expression with 10 ng/ml in CD146-depleted and -enriched hPDL-MSCs. CD146-enriched hPDL-MSCs revealed a 0.89-fold (n.s.) lower *TNFAIP6* gene expression. TNF-α-induced TSG-6 was undetectable in the conditioned medium. These data suggest that the *TNFAIP6* gene and TSG-6 protein expression levels are similar between CD146-depleted and -enriched hPDL-MSCs, independently from the cytokine environment.

## Discussion

Multiple strategies have been developed and tested to increase the therapeutic potential of transplanted MSCs^15^. One of these strategies is to isolate MSC subpopulations with improved properties, such as CD146-expressing MSCs^24^. This subpopulation, obtained from MSCs of various tissues in the human body, has been thoroughly explored in numerous in vitro and in vivo studies^24,27–36^. These studies primarily demonstrated improved proliferation, differentiation, and colony-forming unit capabilities, as well as increased therapeutic efficacy in various disease models. Despite the growing attention on immunomodulatory abilities as key to the therapeutic potential of MSCs^17^, the immunomodulatory activities of CD146-expressing cells have been compared to those of CD146^−^ MSCs in only a limited number of studies, which have mainly reported enhanced immunosuppressive effects^24,30,36^. However, only two studies have investigated the expression of two classical immunomediators in bone marrow-^24^ and adipose tissue-^37^ derived MSCs. Moreover, none of the previous studies addressed the expression of immunomediators under various inflammatory conditions, which is a known determinant of the immunomodulatory abilities of MSCs^14^. Hence, there is currently insufficient information regarding the differences in immunomediator expression between CD146-expressing and non-expressing MSCs. Thus, in this study, we aimed to systematically compare the expression and production levels of IDO-1, CD274/PD-L1, PTGS-2/PGE_2_ and TSG-6 between CD146-expressing and CD146^−^ MSCs isolated from the human PDL.

In the first approach, we compared the protein expression of immunomediators between CD146^+^ and CD146^−^ hPDL-MSCs within heterogenous populations without any prior cell enrichment procedures. The cytokine treatment primarily resulted in significant increases in the expression of IDO-1, PD-L1, and PTGS-2, which were observed in both subpopulations. However, we did not detect any clear or consistent differences in the percentage of immunomediators or their expression levels between CD146^+^ and CD146^−^ hPDL-MSC subpopulations. An increased rate of immunomodulator-positive hPDL-MSCs and protein expression levels in CD146^+^ hPDL-MSCs was only observed in a few cases, including IL-1β and TNF-α-induced IDO-1, or IL-1β, IFN-γ, and TNF-α- induced PD-L1 expression. This higher expression of immunomediators in the CD146^+^ subpopulations may lead to improved immunosuppressive properties.

In contrast, the proportion of TSG-6^+^ hPDL-MSCs was significantly lower in CD146^+^ hPDL-MSCs, both in the absence and presence of all three types of cytokines. The lack of a notable overall increase in TSG-6^+^ hPDL-MSCs with any of the cytokines suggests that the biological relevance of the reduced proportion of TSG-6^+^ within CD146^+^ hPDL-MSCs is eminent only under basal, non-inflammatory conditions. The production of TSG-6 by MSCs is thought to promote the polarization of macrophages toward an anti-inflammatory phenotype^38^. However, how far these differences in TSG-6 expression between CD146^+^ and CD146^−^ MSCs are physiologically relevant is not known. Overall, these findings imply that the variations in immunomediator expression between CD146^+^ and CD146^−^ hPDL-MSCs strongly depend on a combination of various factors, including the type of immunomediator, the inflammatory environment, and the concentrations of cytokines.

The aforementioned conclusion is also supported by using an alternative flow cytometry gating strategy to investigate the true proportion of CD146^+^/immunomediator^+^ hPDL-MSCs within heterogeneous populations. This involved comparing the percentage of CD146^−^ IDO-1^+^ vs. CD146^+^ IDO-1^+^, CD146^−^ PD-L1^+^ vs. CD146^+^ PD-L1^+^, CD146^−^ PTGS-2^+^ vs. CD146^+^ PTGS-2^+^ and CD146^−^ TSG-6^+^ vs. CD146^+^ TSG-6^+^. This showed a significantly higher proportion of CD146^+^ IDO-1^+^, and CD146^+^ PTGS-2^+^ hPDL-MSCs compared to CD146^−^ IDO-1^+^ and CD146^−^ PTGS-2^+^ hPDL-MSCs in the presence of IL-1β or TNF-α and the absence of any cytokine, respectively. The significantly increased percentage of CD146^+^ PTGS-2^+^ hPDL-MSCs in the presence of IFN-γ or TNF-α may have no biological relevance due to the lack of an overall impact of these cytokines on the proportion of PTGS-2^+^ hPDL-MSCs. So far, only Peng et al.^39^ verified the percentage of CD146^+^ PD-L1^+^ and CD146^−^ PD-L1^+^ MSCs isolated from the adipose tissue, showing a higher proportion of CD146^+^ PD-L1^+^ MSCs under basal conditions. In our study, PD-L1 exhibited no significant differences, although the percentage of CD146^+^ PD-L1^+^ also tended to be higher in the absence and presence of various cytokines.

Interestingly, the proportions of the CD146^−^ IDO-1^+^ vs. CD146^+^ IDO-1^+^, CD146^−^ PD-L1^+^ vs. CD146^+^ PD-L1^+^, CD146^−^ PTGS-2^+^ vs. CD146^+^ PTGS-2^+^ and CD146^−^ TSG-6^+^ vs. CD146^+^ TSG-6^+^ populations within the heterogenous hPDL-MSC populations were noticeably smaller than the corresponding percentage of immunomodulator-positive hPDL-MSCs found within both CD146^+^ and CD146^−^ subpopulations. Given that our findings demonstrated higher protein expression of immunomediators in CD146^+^ hPDL-MSCs under specific conditions, it raises the possibility that enriching CD146^+^ MSC subpopulations could enhance the immunomodulatory capabilities of a particular MSC population, thereby increasing its therapeutic potential. Previous studies have already used this strategy, showing increased immunosuppressive activities against T lymphocytes in CD146^+^ MSCs that were isolated by magnetic-activated cell sorter from the bone marrow^24^ and umbilical cord^30,36^.

In the second approach, we used this kind of cell sorting to generate hPDL-MSC populations that were either CD146-depleted or -enriched. We referred to these two populations as “depleted” and “enriched” because both still contained CD146^+^ and CD146^−^ hPDL-MSCs. All three exogenous cytokines caused a significant increase in IDO-1, *CD274*/PD-L1, *PTGS-2*/PGE_2_, and *TNFAIP6*/TSG-6 gene and protein expression. These enhancements were evident in both CD146-depleted and -enriched hPDL-MSC populations. The expression of immunomediators was mostly minimally elevated in CD146-enriched hPDL-MSCs, with significant differences only arising under specific conditions: There was higher PD-L1 protein expression in CD146-enriched hPDL-MSCs in basal conditions and in the presence of IFN-γ or TNF-α. Variations in PGE_2_ levels were observed when exposed to TNF-α or IL-1β, causing either a higher or lower level in CD146-enriched populations, respectively. While there was a trend towards higher PGE_2_levels in CD146-enriched hPDL-MSCs under basal conditions, this was not statistically significant. This tendency aligns with findings from Li et al^37^., who reported significantly higher PGE_2_ levels in CD146^+^ MSCs sourced from adipose tissue. In our study, we did not detect significant differences in IDO-1 and *TNFAIP6*/TSG-6 expression, which supports the results of Bowles et al.^24^, who also found no alterations in IDO-1 activity between primed (IFN-γ + TNF-α) CD146^+^ versus CD146^−^ MSCs isolated from bone marrow. However, under basal conditions, Bowles et al.^24^ observed significantly higher IDO-1 activity in CD146^+^ MSCs, which contrasts with our findings, as well as those of Li et al.^37^, who also noted no significant differences between CD146^+^ and CD146^−^ MSCs form adipose tissue. Overall, these results suggest that immunomediator expression is not universally higher in CD146-enriched MSC populations and that the differences between CD146-depleted and -enriched populations are influenced by the type of immunomediator, the cytokine microenvironment, and the tissue source of the MSCs. It is important to mention that CD146-depleted and -enriched MSC populations were obtained through a positive selection method. Assessing the full impact of the antibody and the entire isolation procedure on hPDL-MSCs remains a challenge.

Taking into account the data from both approaches, we can draw several conclusions: Firstly, the detected increased levels of immunomediators found in CD146-expressing hPDL-MSCs suggest enhanced immunomodulatory activities. Previous research has indicated that CD146^+^ MSCs isolated from bone marrow and umbilical cord exhibit greater immunosuppressive activities against T lymphocytes^24,28,30,36^. However, the observed differences in immunomediator expression between CD146-expressing and non-expressing hPDL-MSCs are closely linked to the cytokine environment, which may also restrict the disparity in their immunomodulatory activities. This assumption is supported by Bowles et al.^24^, who reported that unprimed CD146^+^ MSCs showed a significantly stronger suppression of T lymphocytes than CD146^−^ MSCs. However, this advantage of CD146^+^ MSC subpopulations was not observed in the presence of IFN-γ and TNF-α^24^. Moreover, our data show no clear tendency for higher immunomediator expression in CD146^+^ hPDL-MSCs. Given that the relevance of these immunomediators in their interactions with immune cells differs based on the type of immune cells involved, it is crucial to evaluate the immunomodulatory effects of CD146-expressing versus non-expressing MSCs across various immune cell types in different panels. To our knowledge, current studies have primarily focused on T lymphocytes and PBMCs^24,28,30,36^.

Second, it is uncertain whether CD146 is the ideal surface marker for capturing almost all MSCs expressing immunomediators. Our data demonstrated that the differences in the percentages of immunomediator-expressing cells in both CD146^+^ and CD146^−^ hPDL-MSCs were lower than the absolute percentage of these cells. Moreover, the results showed large proportions of CD146^−^ IDO-1^+^, CD146^−^ PD-L1^+^, CD146^−^ PTGS-2^+^, and CD146^−^ TSG-6 hPDL-MSCs, some of which were comparable to or exceeded the levels found in the corresponding double-positive populations. This was also confirmed by the detected immunomediator production in the CD146-depleted hPDL-MSC population, which was mainly comparable to CD146-enriched hPDL-MSCs or even higher. Hence, it can be assumed that the surface marker CD146 does not completely encompass all immunomediator-expressing hPDL-MSCs and may be insufficient as a marker to fully enrich hPDL-MSCs with the best immunomodulatory potential.

## Methods

### Ethics approval and consent to participate

The third molar extraction procedure was approved by the Ethics Committee of the Medical University of Vienna (EK-Nr. 1079/2019, valid until 03/2026). All participants signed an informed written consent form before the tooth extraction. All experimental procedures were conducted following the Declaration of Helsinki and the Good Scientific Practice Guidelines of the Medical University of Vienna.

### Primary hPDL-MSCs isolation and cultivation

Third molars were extracted from individuals with a healthy periodontium due to orthodontic indications. The participants were aged between 18 and 32. Patients with the following conditions were excluded from the study: < 18 years and > 45 years, periodontal disease, gingival inflammation, periodontal therapy within the last three months, less than 20 teeth, pregnancy or lactation, systemic diseases, acute infections, immunodeficiency, immune suppressive or anti-inflammatory medication, antibiotic therapy, and any radio- or chemotherapy in medical history records.

Primary hPDL-MSCs were isolated from extracted teeth as described in our previous publication^40^. Briefly, the PDL tissue was scratched from the mid-third of the tooth’s roots and chopped, followed by cultivation of the minced tissue in Dulbecco’s Modified Eagles Medium (DMEM; Capricorn Scientific GmbH, Ebsdorfergrund, Germany) supplemented with 4.5 μg/ml glucose, L-glutamine, 10% fetal bovine serum (FBS; Gibco, Carlsbad, CA,USA), 50 μg/ml streptomycin (Gibco, Carlsbad, CA, USA) and 100 U/ml penicillin (Gibco,Carlsbad, CA, USA). The cultured PDL tissue was incubated for several weeks at 37 °C, 5% carbonic acid gas, and 95% humidity until hPDL-MSCs had grown out of the tissue pieces. Every week, the medium was replaced by a fresh one. Outgrown hPDL-MSCs were harvested, expanded, and sub-cultured under the above-described conditions. Passages four to seven were used for experiments.

The MSC nature of hPDL-MSCs was verified following the minimal criteria for MSCs defined by the ISCT^6,7^. The percentage of CD29^+^, CD90^+^, CD105^+^, and CD146^+^ hPDL-MSCs and the absence of the hematopoietic surface markers CD14, CD31, CD34, and CD45 were tested by flow cytometry analysis. Additionally, in vitro-triggered osteogenic differentiation was monitored by Alizarin Red staining. The findings from these verifications have already been reported in our former studies^14,41,42^.

### Immunomediator expression analysis in CD146^-^ vs. CD146^+^ hPDL-MSCs within a population

#### Treatment

hPDL-MSCs were seeded with a density of 2.7 × 10^4^ per cm^2^ in a 6-well plate (TPP, Trasadingen, Switzerland) using 3 ml DMEM supplemented with 10% FBS, 50 μg/ml streptomycin, and 100 U/ml penicillin. After overnight incubation, hPDL-MSCs were stimulated with 1–10 ng/ml TNF-α, 0.5–5 ng/ml IL-1β, or 10–100 ng/ml IFNγ (all from Peprotech, London, United Kingdom). hPDL-MSCs without any exogenous cytokines served as control. For the cell treatment, the medium was changed to 1 ml DMEM supplemented with 50 μg/ml streptomycin, and 100 U/ml penicillin but without any FBS. After 48 h of incubation, CD146/IDO-1, CD146/PD-L1, CD146/TSG-6, or CD146/PTGS-2 co-expression was determined by immunostaining followed by flow cytometry analysis.

#### Flow cytometry analysis

##### CD146/IDO-1, CD146/PD-L1, CD146/TSG-6, and CD146/PTGS-2 co-expression analysis

2.5 × 10^5^ hPDL-MSCs were used for CD146/IDO-1, CD146/PD-L1, CD146/TSG-6, and CD146/PTGS co-immunostaining. Only for CD146/TSG-6 immunostaining, TSG-6 secretion was inhibited by adding 25 µg/ml brefeldin A (Sigma-Aldrich, St. Louis, MO, USA) to the hPDL-MSCs 24 h before immunostaining. After harvesting, hPDL-MSCs were washed with staining buffer including 3% BSA (bovine serum albumin, Capricorn Scientific GmbH, Ebsdorfergrund, Germany) in 1xPBS and 0.09% NaN_3_ (Merck Millipore, Burlington, VT, USA). This was followed by adding 0.125 µg R-phycoerythrin (R-PE) conjugated mouse anti-human CD146 monoclonal (clone P1H12; IgG1, kappa isotype) antibody (Thermo Fisher Scientific, Waltham, MA, USA) in a total of 50 µl staining buffer. For CD146/PD-L1 co-immunostaining, 1 µg PerCP-eFluor™ 710 conjugated mouse anti-human CD274 (PD-L1, B7-H1) monoclonal (clone MIH1; IgG1, kappa isotype) antibody (Thermo Fisher Scientific, Waltham, MA, USA) were additionally added followed by a 30-min incubation time at room temperature. After washing CD146/PD-L1 co-labeled hPDL-MSCs with staining buffer, the cells were resuspended in 0.5 ml staining buffer for flow cytometry analysis.

The Attune™ Nxt Acoustic Focusing flow cytometer (Thermo Fisher Scientific, Waltham, USA) was used to acquire 20,000 cells per sample. All used antibody-fluorophore conjugates were excited with the blue laser at 488 nm and emitted light was detected in the BL1 (PTGS-2-AlexaFluor™ 488, TSG-6-AlexaFluor™ 488), BL2 (CD146-R-PE), and BL3 (PD-L1-PerCP-eFluor™ 710, IDO-1-PerCP-eFluor™ 710) channels. hPDL-MSCs stimulated with 100 ng/ml IFN-γ and 5 ng/ml IL-1β were used as compensation controls for CD146/PD-L1 and CD146/PTGS-2 co-immunostaining, respectively. The AbC™ Total Antibody Compensation Bead Kit (Thermo Fisher Scientific, Waltham, MA, USA) was used as compensation control for CD146/IDO-1 and CD146/TSG-6 immunostaining, following the company’s protocol. The post-acquisition analysis was done by the FCS Express™ software (De Novo Software by Dotmatics, Pasadena, CA, USA). After excluding cell debris and co-incidence events, two different gating strategies were applied (see Supplementary Fig. S3). The first gating approach allowed us to identify the percentage of CD146^+^ immunomediator^+^ and of CD146^−^ immunomediator^+^ hPDL-MSCs within the whole cell population (see Supplementary Fig. S3a). The second gating strategy enabled to determine the % of immunomodulator-positive hPDL-MSCs within the CD146^+^ and CD146^−^ hPDL-MSC population and the MFI of the whole cell population (see Supplementary Fig. S3b). Due to different autofluorescence of the CD146^+^ and CD146^−^ gated hPDL-MSCs within the immunomediator detection channels, CD146 single stained CD146^+^ and CD146^−^ hPDL-MSCs were used as controls for placing the gates in the immunomediator detection channels. The MFI fold-change was calculated by normalizing the MFI to the appropriate CD146 single staining controls (fold-change MFI = 1).

### Immunomediator expression analysis in CD146-depleted/enriched hPDL-MSC populations

#### Depletion and enrichment of CD146^+^ hPDL-MSCs

CD146-depleted and -enriched hPDL-MSC populations were accomplished using the Dynabeads^®^ FlowComp™ Flexi, part A kit, and the unconjugated, monoclonal mouse anti-human CD146 antibody (clone P1H12, IgG1 kappa) following the manufacturer’s instructions (both Thermo Fisher Scientific, Waltham, MA, USA). Before the isolation procedure, the unconjugated, anti-human CD146 antibody was desalted and biotinylated using the Zeba™ Spin Desalting Columns and the DSB-X™ Biotin Protein Labeling Kit (both Thermo Fisher Scientific, Waltham, MA, USA), respectively, following the protocols of the producer. In brief, Zeba™ protein desalting spin columns were prepared by compacting and washing the resin with ultrapure water via several rounds of centrifugation. A low-volume unconjugated anti-human CD146 antibody sample was added to the middle of the compacted resin, followed by ultrapure water as a stacker for maximal antibody recovery. Desalted unconjugated anti-human CD146 antibody was collected by centrifugation.

0.2 ml desalted antibody (0.5 mg/ml) was mixed with freshly prepared 1 M NaHCO_3_ and 2 µl DSB-X™ biotin succinimidyl ester (in dimethyl sulfoxide) solution followed by stirring for 3 h at room temperature. The spin column was prepared with approximately 1.5 ml of purification resin. After settling the resin, the column buffer was drained from the spin column, and 0.2 ml of the reaction mixture was loaded onto the center of the spin column and incubated until it was absorbed by the resin bed. The DSB-X™ biotin-conjugated anti-human CD146 antibody was collected by centrifugation in 0.2 ml PBS (phosphate buffered saline; pH 7.2 and 2 mM NaN_3_). Bovine serum albumin (BSA; Capricorn Scientific GmbH, Ebsdorfergrund, Germany) was added to reach a final BSA concentration of 1 mg/ml for storage at 4 °C. The antibody concentration was estimated to be 0.42 mg/ml using the following equation:

$$\:\frac{inital\:mg\:of\:antibody\:*\:0.85}{ml\:in\:collection\:tube}=mg/mlDSBX^{\mathrm{TM}}\:biotin\:labeled\:antibody$$

For the depletion and enrichment of CD146^+^ hPDL-MSCs (see Supplementary Fig. S1b), 7.9 × 10^7^ FlowComp™ Dynabeads^®^ with an initial concentration of ~ 1.5 × 10^9^ beads/ml were washed with isolation buffer (Ca^2+^ and Mg^2+^-free phosphate buffer saline buffer (Gibco, Carlsbad, CA, USA) complemented with 2mM ethylendiamintetraacetat (Merck Millipore, Burlington, VT, USA) and 2% FBS). After placing the bead solution in a DynaMag™ magnet, the isolation buffer was discarded, and the FlowComp™ Dynabeads^®^ were dissolved in fresh isolation buffer, reaching the initial bead concentration per ml.

After harvesting hPDL-MSCs that have not exceeded 80% confluency, 5 to 10 × 10^6^ hPDL-MSCs were washed with cold isolation buffer. 12.5 µl DSB-X™ biotinylated anti-human CD146 antibody was added to 100 µl hPDL-MSCs, which were incubated for 30 min at 4 °C. After washing hPDL-MSCs with cold isolation buffer, hPDL-MSCs were resuspended in 1 ml cold isolation buffer, adding 7.9 × 10^7^ FlowComp™ Dynabeads^®^ to get a final concentration of ~ 8 FlowComp™ Dynabeads^®^ per hPDL-MSCs. An incubation time of 30 min with rolling and tilting at 4 °C was followed by adding isolation buffer and putting the cell suspension into the DynaMag™ magnet. The non-target cells (CD146-depleted hPDL-MSC fraction) were harvested by tilting the DynaMag™ magnet after 2 min of incubation, which was repeated three times in total, each time with a fresh isolation buffer. The target cells (CD146-enriched hPDL-MSC fraction) were resuspended in FlowComp™ Release buffer and incubated for 10 min at 4 °C with rolling and tilting. Subsequently, the CD146-enriched hPDL-MSCs population was resuspended and placed in the DynaMag™ magnet for one minute. The supernatant with the FlowComp™ Dynabeads^®^-free hPDL-MSCs was harvested by tilting the DynaMag™ magnet, which was repeated twice. The CD146-depleted and -enriched hPDL-MSC populations were immediately resuspended in DMEM, supplemented with 4.5 g/ml glucose, L-glutamine, 10% FBS, 50 µg/ml streptomycin and 100 U/ml penicillin and were cultured under the same conditions as previously.

The CD146-depleted and -enriched fractions were immediately verified after the isolation procedure. 2 × 10^4^ to 1 × 10^5^ hPDL-MSCs from each fraction were stained with the secondary R-PE goat-anti-mouse IgG1 antibody (1:800; Thermo Fisher Scientific, Waltham, MA, USA) for 30 min at room temperature in staining buffer containing 3% BSA (Capricorn Scientific GmbH, Ebsdorfergrund, Germany) in 1xPBS and 0.09% NaN_3_ (Merck Millipore, Burlington, VT, USA). After washing hPDL-MSCs with staining buffer, hPDL-MSCs were resuspended in 0.5 ml staining buffer for flow cytometry analysis, using the Attune™ Nxt Acoustic Focusing flow cytometer (Thermo Fisher Scientific, Waltham, MA, USA). The R-PE fluorophore was excited with the blue laser at 488 nm, and emitted light was detected in the BL2 channel. hPDL-MSCs only labeled with the primary anti-human CD146 antibody served as control. After excluding co-incidence events and cell debris, the percentage of CD146^+^ hPDL-MSCs was determined in the CD146-depleted and -enriched hPDL-MSCs populations.

#### Treatment of CD146-depleted and -enriched hPDL-MSC populations

2.7 × 10^4^ cells from the CD146-depleted and -enriched hPDL-MSC populations were seeded per cm^2^ in separated wells. For the gene expression analysis of *IDO-1*,* CD274*,* PTGS2*, and *TNFAIP6*, hPDL-MSCs were seeded in 24-well plates (TPP, Trasadingen, Switzerland) using 0.5 ml DMEM per well, supplemented with 10% FBS, 50 µg/ml streptomycin, and 100 U/ml penicillin. For IDO-1, PD-L1, and TSG-6 protein expression and PGE_2_ production analysis, 6-well plates were used, seeding 2.5 x10^5^ hPDL-MSCs in 3 ml DMEM per well as described above. After 24 h of incubation, hPDL-MSCs were treated with 1–10 ng/ml TNF-α, 0.5-5 ng/ml IL-1β, or 10–100 ng/ml IFN-γ for additional 48 h. 0.5 ml and 1 ml DMEM supplemented with 50 µg/ml streptomycin, 100 U/ml penicillin, and without FBS were used for stimulation in the 24- and 6-well plate format, respectively. hPDL-MSCs were harvested from the 24-well plates and lysed for gene expression analysis, whereas hPDL-MSCs from 6-well plates were used to investigate IDO-1 and PD-L1 protein expression. Conditioned media from 6-well plates were harvested for TSG-6 and PGE_2_ secretion and IDO-1 enzymatic activity analysis.

#### Gene and protein expression analysis

##### IDO-1, CD274, PTGS2, and TNFAIP6 gene expression analysis

5 × 10^4^ CD146-depleted and -enriched hPDL-MSCs were used per sample for gene expression analysis. Cell lysis, reverse transcription, and quantitative polymerase chain reaction (qPCR) were conducted using the TaqMan™ Gene Expression Cells-to-C_t_™ kit (Thermo Fisher Scientific, Waltham, MA, USA) according to the manufacturer’s guidelines. After the lysis, complementary deoxyribonucleic acid (cDNA) was generated. The Biometra TOne Serie thermocycler (Analytik Jena An Endress + Hauser company, Jena, Germany) was used to heat cell lysates to 37 °C for 1 h, followed by 5 min at 95 °C. qPCR was performed using the QuantStudio™ 3 Real-Time PCR System (Thermo Fisher Scientific, Waltham, MA, USA). Synthesized cDNA was pre-heated to 95 °C for 10 min, followed by heating the cDNA to 95 °C for 15 s and 60 °C for one minute for a total of 50 cycles. At the end of each cycle, the fluorescence emission from the TaqMan^®^ probes was detected using the FAM-MGB dye-quencher combination. All target genes were amplified and detected in paired reactions using the following TaqMan^®^ assays (all from Thermo Fisher Scientific, Waltham, MA, USA): Hs00984148_m1 (*IDO-1*), Hs00204257_m1 (*CD274*), HS00153133_m1 (*PTGS2*), Hs00200180_m1 (*TNFAIP6*), and Hs99999905_m1 (*GAPDH*). The housekeeping gene glyceraldehyde-3-phosphate dehydrogenase (*GAPDH*) was used as endogenous control. The determined cycle threshold (C_t_) values were normalized to the appropriate *GAPDH*-associated C_t_ values (ΔC_t_) followed by normalization to the unstimulated controls (ΔΔC_t_) within the CD146-depleted and -enriched populations. The n-fold expression was calculated using the 2^−ΔΔCt^ equation compared to the appropriate unstimulated controls (n-fold expression = 1).

##### IDO-1 and PD-L1 protein expression analysis

2.5 × 10^5^ CD146-depleted and -enriched hPDL-MSCs were used for intracellular IDO-1 and PD-L1 surface marker single staining. After harvesting, hPDL-MSCs were washed with the staining buffer containing 3% BSA and 0.09% NaN_3_ in 1xPBS. For intracellular IDO-1 staining, hPDL-MSCs were fixed and permeabilized using the eBioscience™ Intracellular Fixation & Permeabilization Buffer Set following the company’s instructions. IDO-1 was stained by 0.06 µg R-PE conjugated mouse anti-human IDO-1 monoclonal (clone eyedio; IgG1, kappa isotype) antibody (Thermo Fisher Scientific, Waltham, MA, USA) in a total of 50 µl eBioscience™ 1x Permeabilization buffer. PD-L1 surface marker was stained without fixation and permeabilization using 0.5 µg R-PE conjugated mouse anti-human CD274 (PD-L1, B7-H1) monoclonal (clone MIH1; IgG1 kappa isotype) antibody (Thermo Fisher Scientific, Waltham, MA, USA) in 50 µl staining buffer. After 30 min of incubation at room temperature, stained IDO-1 or PD-L1 stained hPDL-MSCs were washed with 2 ml eBioscience™ 1x Permeabilization buffer or staining buffer, respectively. For analysis, stained hPDL-MSCs were resuspended in 0.5 ml staining buffer and a maximum of 20,000 cells were acquired by the Attune™ Nxt Acoustic Focusing flow cytometer. The blue laser at 488 nm exited the R-PE fluorochrome, and emitted light was detected in the BL2 channel. Since the BL-2 channel-associated autofluorescence of the CD146-depleted hPDL-MSCs was higher compared to the CD146-enriched hPDL-MSCs population (see Supplementary Fig. S4 online), unstained CD146-depleted and -enriched hPDL-MSCs were used as negative controls, placing the gates by the appropriate unlabeled control for the CD146-depleted and -enriched samples, respectively. After excluding cell debris and co-incidence events, the percentage of IDO-1 or PD-L1 positive hPDL-MSCs and the median fluorescence intensity (MFI) of the whole cell population were determined. The MFI fold-change was calculated by normalizing the MFI to the appropriate unstained controls (fold-change MFI = 1). The post-acquisition analysis was performed by the FCS Express™ 7 software.

##### IDO-1 enzymatic activity analysis

Conditioned medium from the CD146-depleted and -enriched hPDL-MSCs was harvested and 2:1 diluted with 30% trichloroacetic acid (Sigma-Aldrich, St. Louis, MO, USA). After a 30-min incubation time at 65 °C and a centrifugation step, the supernatant was mixed 1:1 with Ehrlich’s reagent (20 mg/ml p-dimethylbenzaldehyde diluted in glacial acetic acid; Sigma-Aldrich, S. Louis, MO, USA). Following an incubation time of 10 min at room temperature on a shaker, the absorbance was photometrically measured in duplicates at 492 nm using the BioTek Synergy HTX Multimode Reader (BioTek Instruments, Inc., Winooski, VT, USA). To determine the L-kynurenine concentrations, the measured optical density (OD_492_) values were plotted against a standard curve (linear regression fit) with known L-kynurenine concentrations ranging between 7.8 μm and 1000 μm. DMEM without FBS served as blank. The determined L-kynurenine concentrations were normalized to the total cell number per sample, which was calculated using the Neubauer-improved cell counting chambers (NanoEnTek, Soul, South Korea) after harvesting the conditioned medium.

##### TSG-6 protein expression and PGE_2_ production analysis

Conditioned medium from the CD146-depleted and -enriched hPDL-MSCs was collected and stored at − 80 °C. TSG-6 protein levels and PGE_2_ levels were determined using the RayBio^®^ Human TSG-6 enzyme-linked immunosorbent assay (ELISA) kit (RayBiotech, Peachtree Corners, GA, USA) and the Prostaglandin Es Parameter assay kit (R&D Systems, Minneapolis, MN, USA), respectively. To determine TSG-6 and PGE_2_ levels, the absorbance was measured at 450 nm using the BioTek Synergy HTX Multimode Reader (BioTek Instruments, Inc., Winooski, VT, USA). For the PGE_2_ parameter assay, wavelength correction was performed by measuring the absorbance at 540 nm, subtracting the values from the OD_450_, and subtracting the non-specific binding control value. The determined OD_450_ values were plotted against a standard curve with known TSG-6 and PGE_2_ concentrations, ranging from 0.205 ng/ml to 50 ng/ml and 30 pg/ml to 2500 pg/ml, respectively.

### Statistical analysis

All data were received from at least five repetitions using hPDL-MSCs isolated from at least five different individuals. For statistical analysis, the SPSS Statistics software (version 26.0, IBM, Armonk, NY, USA) was used. Normal distribution of the data was verified by the Kolmogorov–Smirnov-Test. Due to paired and non-parametric data, the Friedmann Test for multiple comparisons, followed by the Wilcoxon Test for pairwise comparisons, was performed for all data. The statistical significance was defined by p-values < 0.05. P-values ≥ 0.05 are marked as not significant (n.s.) in the results section.

## Supplementary Information

Below is the link to the electronic supplementary material.

Supplementary Material 1

## Data Availability

All data which support the conclusion are shown in the manuscript. The datasets generated and analyzed during the current study are available from the corresponding author on reasonable request.
