# Supplementary material for: Immunomediator expression in human periodontal ligament MSCs varies depending on surface CD146 expression
Source: Sci Rep. 2026 Feb 22;16:10195. doi: 10.1038/s41598-026-38627-z (PMC13022347; doi:10.1038/s41598-026-38627-z)
Supplement: Supplementary file 1 — Supplementary Material 1 [file 41598_2026_38627_MOESM1_ESM.docx]

**Supplementary Information**

**Immunomediator expression in human periodontal ligament MSCs varies depending on surface CD146 expression**

Behm Christian^1^, Miłek Oliwia^1^, Schwarz Katharina^1^, Kovar Alexander^1^, Andrukhov Oleh^1*^

^1^Competence Center for Periodontal Research, University Clinic of Dentistry, Medical University of Vienna, Sensengasse 2A, 1090 Vienna, Austria

^*^Correspondence to Andrukhov Oleh (oleh.andrukhov@meduniwien.ac.at)


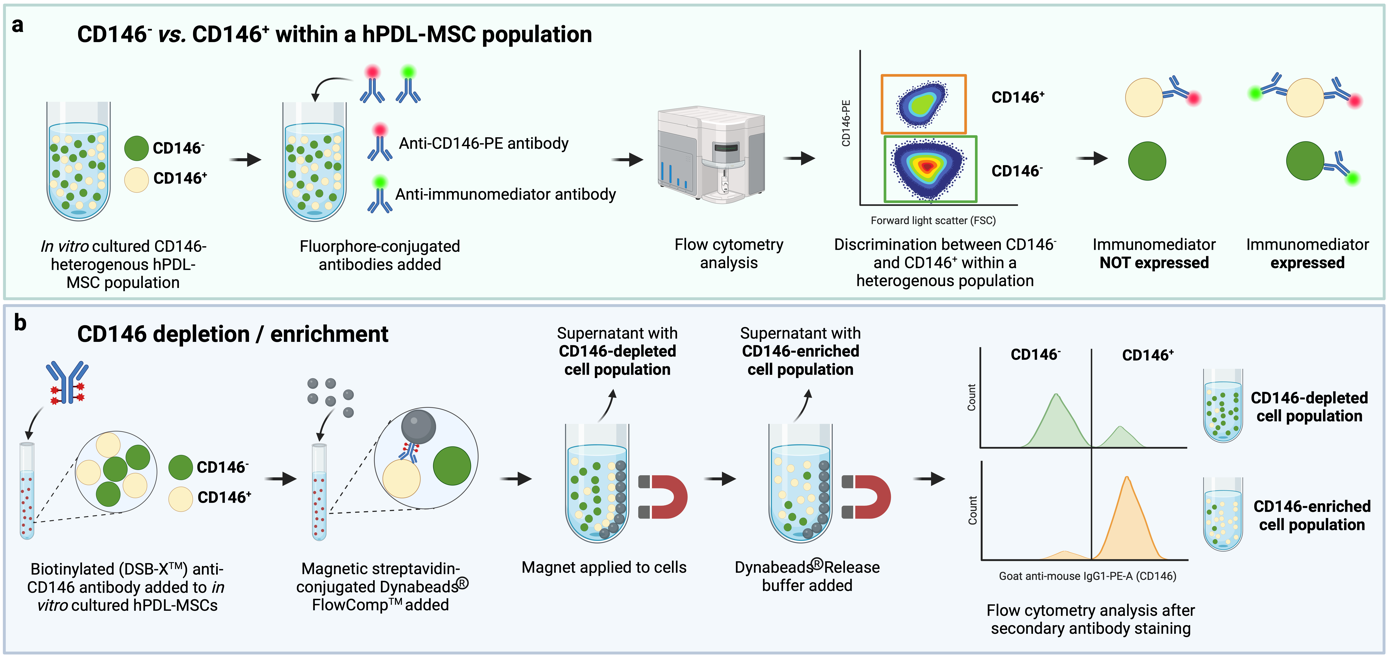


**Supplementary Figure S1.** CD146^-/+^ cells within a hPDL-MSC population *vs.* CD146-depleted and -enriched hPDL-MSCs. The immunomediator expression in hPDL-MSCs was investigated using two different approaches. In the first approach, the immunomediator expression was compared between CD146^-^ and CD146^+^ hPDL-MSCs within a heterogenous (CD146^-/+^) population **(a)**. The second approach used CD146-depleted and -enriched hPDL-MSC populations achieved by a magnetic bead enrichment procedure **(b)**. Created in BioRender. Behm, C. (2025) https://BioRender.com/h85h350

**
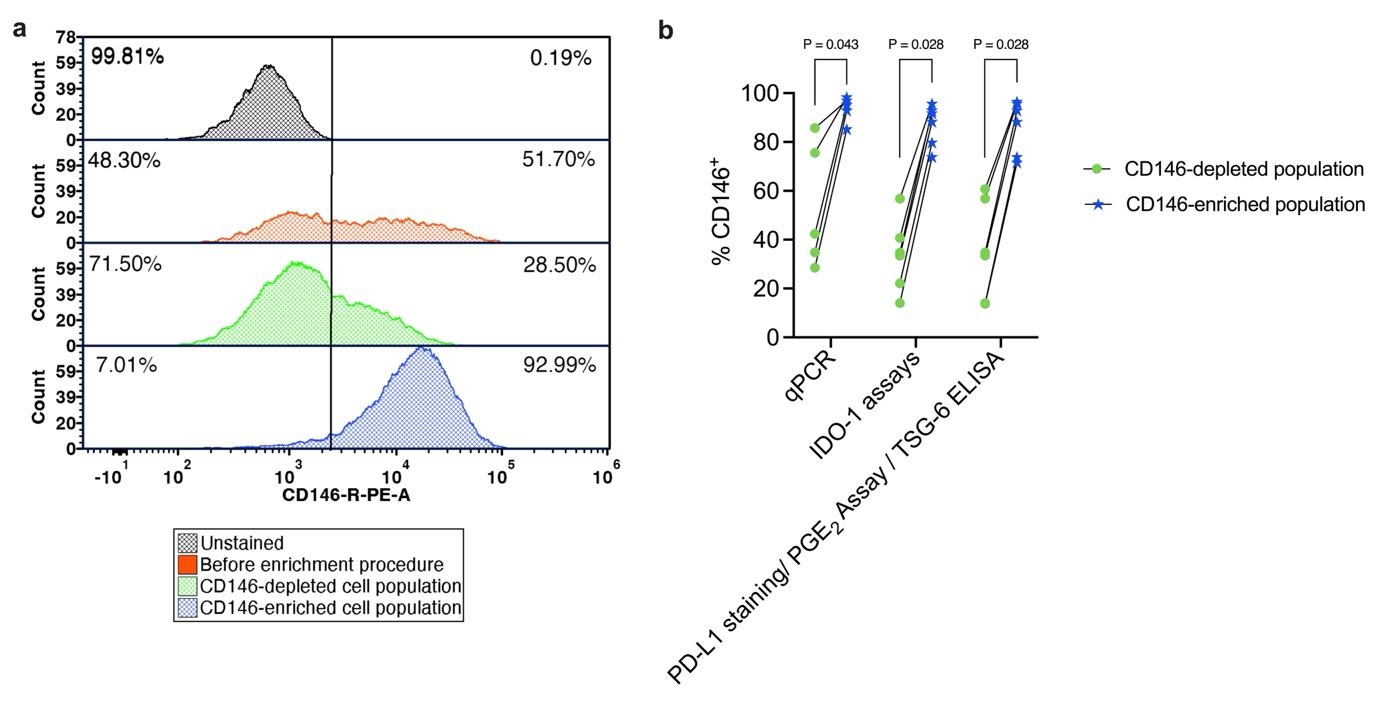
Supplementary Figure S2.** Flow cytometry verification of the depletion and enrichment of CD146^+^ hPDL-MSCs by using Dynabeads®. The % of CD146^-^ and CD146^+^ hPDL-MSCs before the magnetic bead sorting and within the CD146-depleted and -enriched cell populations are displayed as representative histograms **(a)**. After the magnetic bead sorting, the % of CD146^+^ hPDL-MSCs within the depleted- and enriched populations was verified and displayed per cell donor. CD146-depleted and -enriched cell populations from different sorting procedures were used for qPCR, IDO-1 immunostaining / IDO-1 enzymatic activity assay, and PD-L1 immunostaining / PGE_2_ parameter assay / TSG-6 ELISA. The % of CD146^+^ hPDL-MSCs are shown as individual data points. The Wilcoxon Test for pairwise comparison was used to determine statistically significant differences **(b)**.


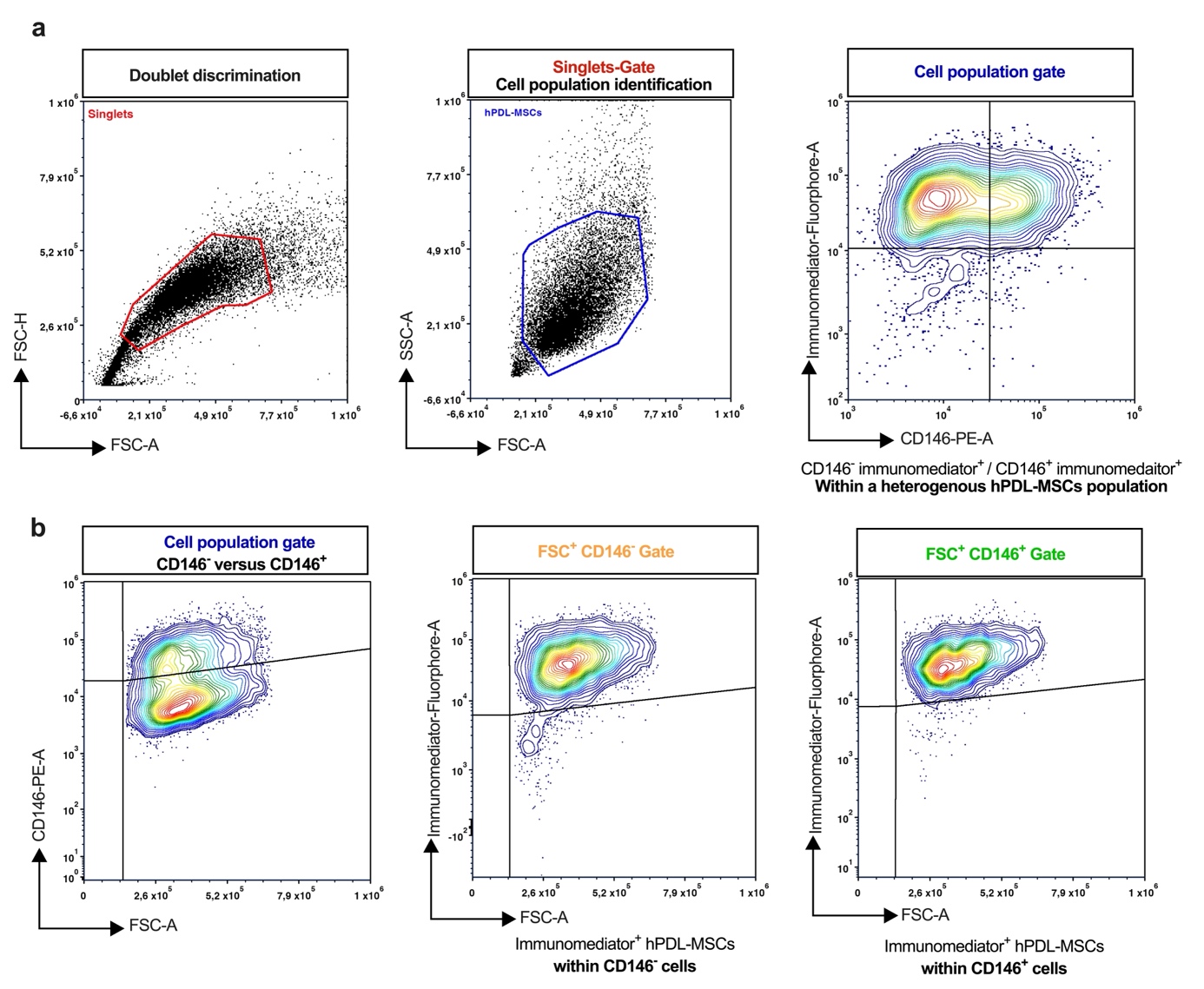
**Supplementary Figure S3.** Two distinct gating approaches to uncover the immunomediator protein expression in CD146^-^ and CD146^+^ cells within a heterogenous hPDL-MSC population. One gating strategy identified the immunomediator single expression and immunomediator/CD146 co-expression in relation to the whole heterogenous population **(a)**. The second gating strategy revealed the immunomediator expression within the CD146^-^ and CD146^+^ hPDL-MSC populations **(b)**. Immunomediator single-labeled and CD146 single-labeled controls were used to place the appropriate quadrant gates.


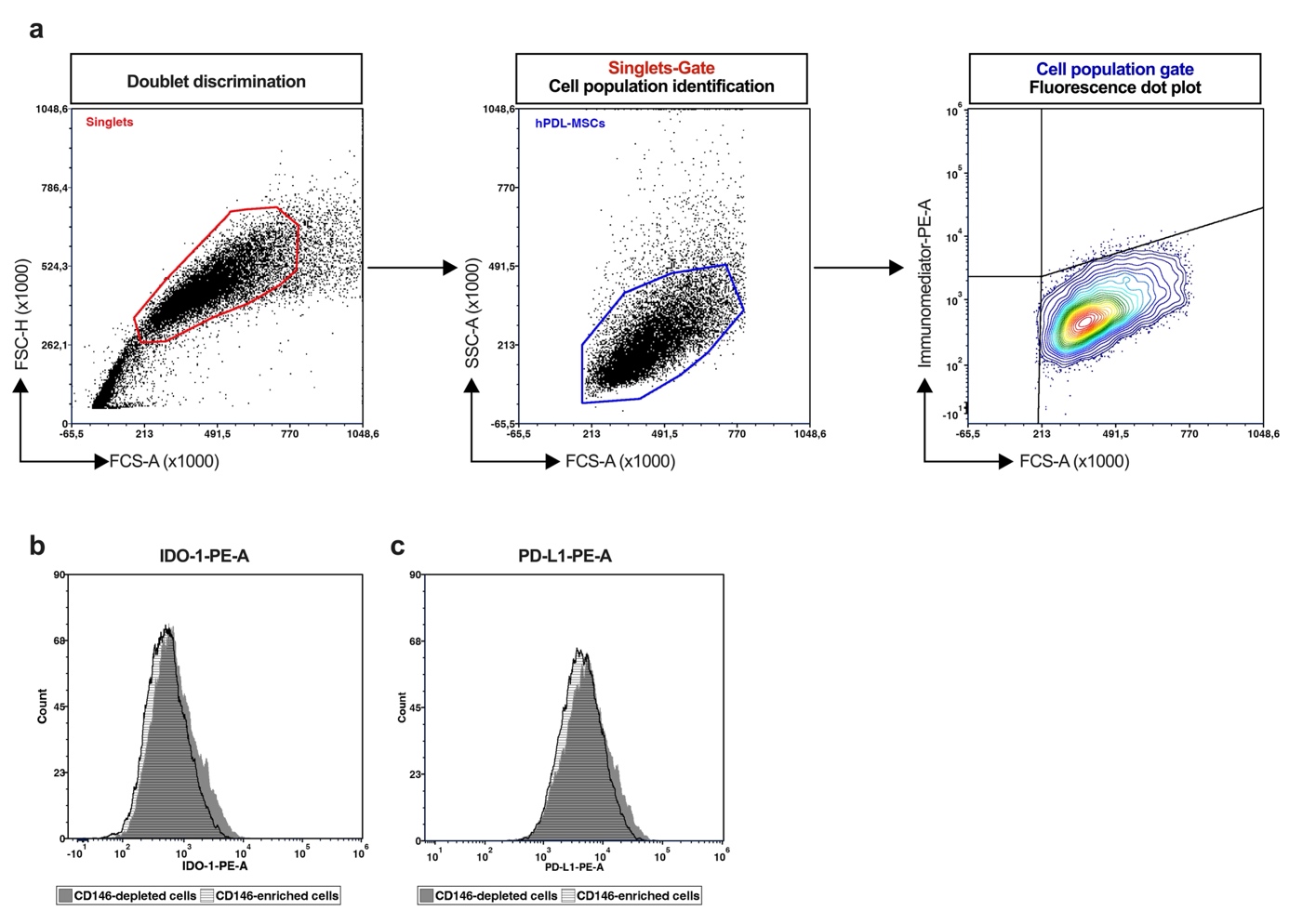
**Supplementary Figure S4.** IDO-1 and PD-L1 protein expression evaluated in CD146-depleted and -enriched hPDL-MSCs using flow cytometry analysis. The gating strategy contained the exclusion of coincidence events and a cell population gate on the morphology plot. The quadrant gate was placed using unlabeled CD146-depleted or -enriched hPDL-MSC populations **(a)**. The histograms demonstrate the different background signals of the CD146-depleted and -enriched hPDL-MSC populations in the R-PE channel **(b-c)**.
